# Estimating the cost-effectiveness of a sequential pneumococcal vaccination program for adults in Germany

**DOI:** 10.1371/journal.pone.0197905

**Published:** 2018-05-24

**Authors:** Ulrike Kuchenbecker, Daniela Chase, Anika Reichert, Julia Schiffner-Rohe, Mark Atwood

**Affiliations:** 1 Xcenda GmbH, Hannover, Germany; 2 INAV GmbH, Berlin, Germany; 3 Pfizer GmbH, Berlin, Germany; 4 PAI, Boston, United States of America; University of Washington, UNITED STATES

## Abstract

**Introduction:**

In Germany, a 23-valent polysaccharide pneumococcal vaccine (PPSV23) is recommended for elderly (60+) and patients 16+ with chronic diseases not associated with immune suppression. For all other patients at risk, sequential immunization with a 13-valent pneumococcal conjugate vaccine (PCV13) first, followed by PPSV23 is recommended. Repeated vaccination with PPSV23 is recommended every 6 years after individual assessment by the physician. This was adopted into the vaccination directive with binding reimbursement and funding. However, additional voluntary services allow statutory health insurances to differentiate from each other. Aim of this study is to estimate the cost-effectiveness of voluntary service scenarios compared to the strategy in place to support informed decision making.

**Methods:**

A microsimulation framework with Markov-type process of a population susceptible to pneumococcal disease over a lifetime horizon was developed to compare effectiveness and cost-effectiveness of different vaccination strategies. We simulated 1,000 iterations for seven scenarios. Assumptions were derived from published literature and probabilistic sensitivity analysis was run to show the robustness of the model.

**Results:**

Our study indicates that all voluntary service strategies could prevent further clinical cases compared to the existing policy. Depending on the scenario, 48–142 invasive pneumococcal disease (IPD), 24,000–45,000 hospitalized all-cause nonbacteremic pneumonia (NBP), 15,000–45,000 outpatient NBP cases, and 4,000–8,000 deaths could be avoided on average. This refers to potential savings of €115 Mio. - €187 Mio. for medical and non-medical costs. Additional costs per patient for the payer are €2.48 to €7.13 and for the society €2.20 to €6.85. The ICER per LYG ranged from €3,662 to €23,061 (payer) and €3,258 to €29,617 (societal). All but one scenario was cost-effective in ≥60% of the generated 1,000 simulations.

**Conclusion:**

Compared to the vaccination strategy in place, the different hypothetical scenarios can be considered cost-effective and suitable as additional voluntary services.

## Introduction

While *Streptococcus pneumoniae* belongs to the most important pathogens causing both invasive (meningitis and bacteremia) and non-invasive (especially pneumonia and acute otitis media) diseases in infants and adults, non-bacteremic pneumonia is by far the most frequent clinical presentation in adults.

Overall, the incidence of pneumococcal disease is much higher for the very young (<2 years) and the elderly (≥65 years), in patients with comorbidities or defects in immune defense [1]. The estimated annual mortality due to pneumococcal diseases in the elderly is 25/100,000 [2]. According to SURVSTAT from the Robert Koch Institute (RKI) the number of observed cases of invasive pneumococcal diseases (IPD) in Germany for adults in all age groups is constantly increasing with a few variations, resulting in increasing overall estimated incidence (all age groups) of 0.45 in 2010 to 0.78 in 2016 per 100,000 population. Especially the reported cases for the elderly (60+) has nearly doubled [3]. The surveillance system of the RKI “PneumoWeb” reported over 1,000 IPD cases in the elderly (60+) in 2016 [4]. Estimation of inpatient cases of all-cause nonbacteremic pneumonia (NBP) in the years 2010–13 shows a mean annual incidence of 178 hospitalized cases of pneumococcal pneumonia per 100,000 population in age group ≥60y. There was an increase of 17% from 2010 to 2013.[2]

Without an explicit vaccination rate defined to reach the target of the pneumococcal vaccination, i.e. to reduce the number of IPD and NBP as well as related sequelae [1], adult pneumococcal vaccination rate in Germany is still suboptimal. According to an analysis of the DEGS1 sample [5], 31.4% of the overall population aged 65–79 years have ever received a pneumococcal vaccination. Currently there are two vaccines indicated for prevention of pneumococcal disease, a 23-valent polysaccharide vaccine (PPSV23) and a 13-valent conjugate vaccine (PCV13) in adults. Since August 2016, a new vaccination recommendation for Germany was published by the German standing committee for vaccination (STIKO): PPSV23 is recommended for all elderly (60+) and all patients 16+ with at least one chronic disease not associated with immune suppression. For all other patients at risk (high-risk representing (congenital or acquired) immunocompromised/immunosuppressed patients, with or without chronic medical conditions), sequential immunization with PCV13 first is recommended, followed by PPSV23. Repeated vaccination with PPSV23 is recommended for patients in all risk groups. Elderly are recommended revaccination every 6 years with PPSV23 following individual assessment by the physician. This recommendation was based on systematic reviews for elderly and patients at risk and supported by a dynamic transmission model for elderly [1, 2]. The STIKO-recommendation was adopted into a new vaccination directive by the Federal Joint Committee in May 2017, defining obligatory reimbursement and funding of pneumococcal vaccination in Germany. Besides obligatory services, statutory health insurances can differentiate themselves by offering additional voluntary services to their insured (Social Law Book V chapter 1, §20i (2)) [6]. Typical additional voluntary services include homeopathic and osteopathic treatment, dental care prophylaxis not covered by obligatory services but also several vaccinations not covered by the STIKO recommendation and therefore not covered by obligatory reimbursement. Beyond this background and combined with the financial impact of the vaccination as well as increasing budgetary pressure, there is a need for a comprehensive evaluation to support an informed decision. To explore the impact of the current vaccination strategy compared to alternative hypothetical ones and to account for uncertainties, microsimulation is required.[7]

Therefore the aim of this study is to simulate different scenarios of additional voluntary vaccination services in terms of cost-effectiveness. This is the first evaluation of the current setting compared to seven different, hypothetical strategies for the German health care setting.

## Materials and methods

### Model design

**Fig 1:**
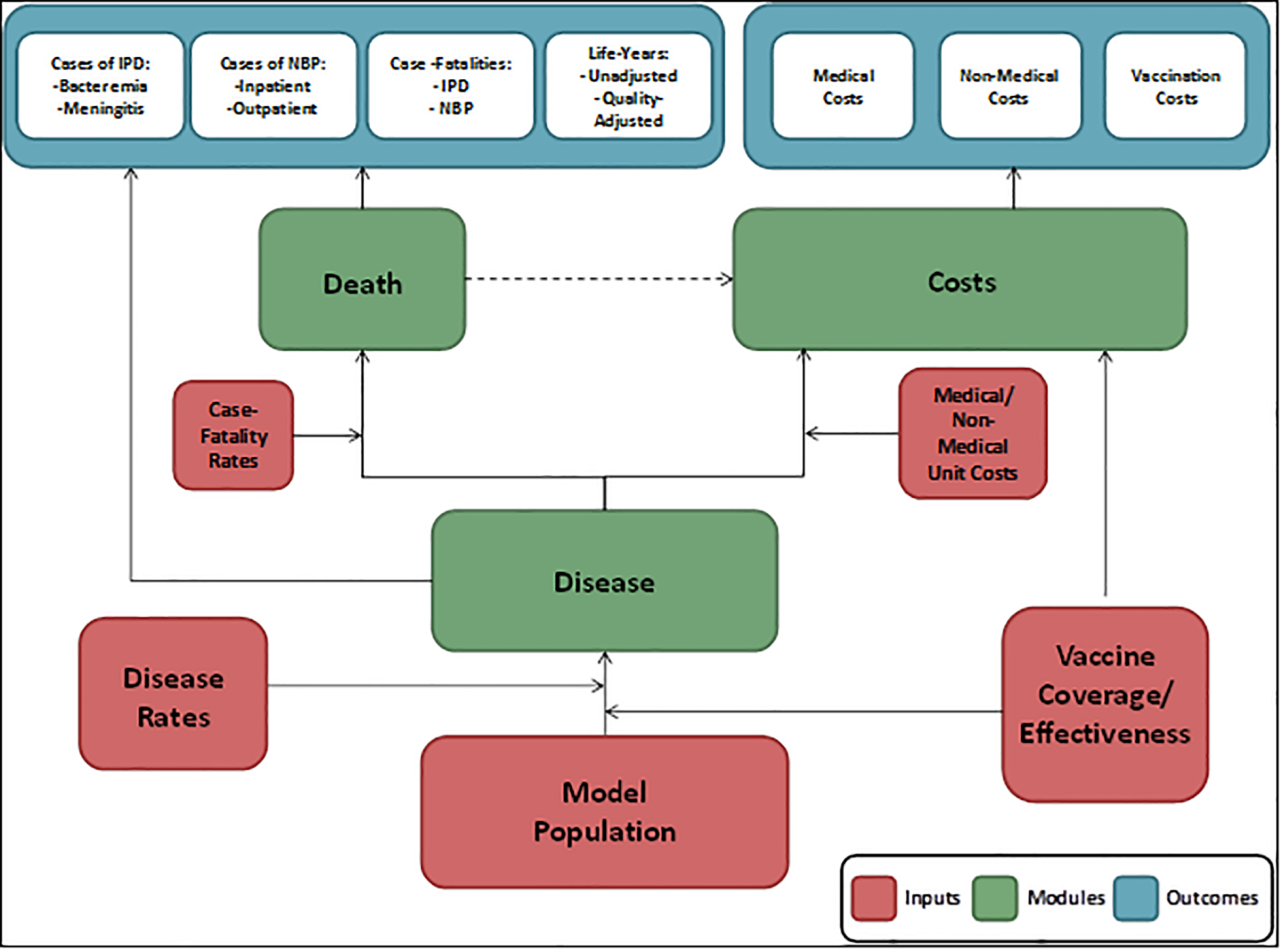
Model structure.

We adapted an existing US model [8, 9] which utilizes a microsimulation framework and a Markov-type process to depict lifetime risks and costs of IPD and NBP, as well as the expected impact of different vaccination schemes, in a hypothetical population of German adults. The model was developed as a static incidence model according to the published health economic guidelines [10, 11]. It simulates the lifetime effects of the current vaccination strategy as recommended by STIKO, compared to a hypothetical strategy from the perspective of the Statutory Health Insurance (SHI, payer) as well as the societal perspective. Finally, the model estimates the cost-effectiveness of the current vaccination strategy compared to hypothetical strategies. Fig 1 illustrates the basic structure of the model.

The target population of the model is the German population aged 18 years and older, separated by their underlying risk of developing pneumococcal diseases and eligible for vaccination against pneumococcal diseases. Depending on the investigated vaccination scenarios (see section “Vaccination strategy”), subjects may receive PPSV23, PCV13, or sequential vaccination (i.e. PCV13 followed by PPSV23); vaccine coverage may vary by age and risk profile. Each incident cohort runs through the model simulating the progression of disease comparing the current vaccination strategy recommended by STIKO and hypothetical strategies. The model tracks a cohort of adults until death or 100 years of age. The cycle length of the model was 1 year so that the seasonality of pneumococcal diseases did not have to be reflected in the model [12]. All parameter values (clinical and economic) are taken directly from the literature, as far as available. Values used by the model “anchored” at the midpoint of their respective age/risk group. We inter-/extrapolated these values linearly between successive points for each year of age for use in the model calculation. Expected clinical and economic outcomes are calculated for each subject annually based on age and risk profile, vaccination status, vaccine type, and time since vaccination.

### Population at model entry

All estimates for the population size, disease rates and case-fatality come from literature and official data (see Table 1). The model population is initially characterized in one-year age increments and by risk profile (i.e., low-, moderate-, or high-risk of pneumococcal disease and/or associated complications). The risk groups were categorized in low, moderate and high-risk. Low-risk (LR) is specified as immunocompetent patients without any chronic medical conditions according to STIKO, moderate-risk (MR) describes immunocompetent patients with at least one chronic medical condition according to STIKO and high-risk (HR) represent immunocompromised/immunosuppressed patients, with or without chronic medical conditions (congenital or acquired). Due to the lack of age-specific data on risk distribution, we assumed an age-independent relation of moderate to low-risk and high to low-risk, respectively.

**Table 1:** Age and risk level specific demographic and epidemiological parameters.

	Age/Risk Profile															
	18–49 yrs.			50–59 yrs.			60–64 yrs.			65–74 yrs.			75–99 yrs.			Source
	LR	MR	HR	LR	MR	HR	LR	MR	HR	LR	MR	HR	LR	MR	HR	
**Population by risk (%)**	75.81	20.97	3.22	60.19	32.87	6.94	48.00	40.63	11.37	38.04	44.26	17.7	25.23	48.12	26.66	[13]
**Incidence per 100,000 persons**																
Bacteremia	0.75	4.68	12.81	1.04	6.51	17.81	0.33	2.07	5.67	6.87	14.98	80.39	3.09	6.73	36.13	[14–16]^a^
Meningitis	0.14	0.88	2.40	0.07	0.43	1.19	0.02	0.14	0.38	0.18	0.40	2.14	0.08	0.18	0.96	
All-cause NBP (hospital.)	44.01	169.48	101.43	76.92	296.18	177.26	84.03	323.57	193.66	243.77	938.69	561.81	1,059.81	4,081.01	2,442.51	[14, 17–19]^a^
All-cause NBP (outpatient)	62.35	240.10	143.70	108.97	419.60	251.13	119.04	458.40	274.35	345.35	1,329.85	795.92	1,501.44	5,781.62	3,460.33	
**Mortality per 100 persons**																
General population	0.10	0.10	0.10	0.47	0.47	0.47	0.89	0.89	0.89	1.75	1.75	1.75	7.30	7.30	7.30	[14]
Bacteremia	5.40	18.20	15.40	5.40	18.20	15.40	5.40	18.20	15.40	29.10	33.00	29.90	29.10	33.00	29.90	[16]
Meningitis	5.40	18.20	15.40	5.40	18.20	15.40	5.40	18.20	15.40	29.10	33.00	29.90	29.10	33.00	29.90	
All-cause NBP (hospital.)	2.10	2.72	4.51	5.66	7.33	12.16	7.65	9.91	16.44	9.40	12.17	20.19	13.34	17.27	28.64	[17]^a^
All-cause NBP (outpatient)	0.00	0.00	0.00	0.00	0.00	0.00	0.00	0.00	0.00	0.00	0.00	0.00	0.00	0.00	0.00	

NBP = nonbacteremic pneumonia | LR = low-risk | MR = moderate-risk | HR = high-risk.

^a^ Calculated values based on these references.

Note: Low-risk is specified as immunocompetent patients without any chronic medical conditions, moderate-risk describes immunocompetent patients with at least one chronic medical condition and high-risk represent immunocompromised/immunosuppressed patients, with or without chronic medical conditions (congenital or acquired).

#### Population size

The size of our hypothetical cohort is based on data of the German adult population aged 18 years and older, separated by their underlying risk of developing pneumococcal diseases. The information were taken from official data from the Federal Health Monitoring (reference year: 2012) [14]. The proportion of people with low, moderate and high-risk for pneumococcal disease was based on an unpublished report using data from the InGef (former Health Risk Institute) related to the publication of Pelton et al. 2015 [13, 20].

#### Disease rates

Data on incidence rates for IPD were based on the publication of Reinert et al. (2005) [15]. A simple extrapolation was done to generate values for the overall population by applying the incidence rate on data from Federal Health Monitoring [14] including a risk adjustment according to data from Hoek et al. (2012) [16]. NBP incidence rates are derived from Schnoor et al. (2007) [19] (in- and outpatient cases) and applied to age groups according to data from the AQUA Institut [18]. Results are adjusted for hospitalization rate based on AQUA Institut [18] and for risk on basis of data from Ewig et al. (2009) [17]. In our model, IPD is reported as combined bacteremia and meningitis outcomes, whereas all-cause NBP is reported by setting of care (inpatient vs. outpatient). Subjects vaccinated (at any time) may be at lower risk of future IPD and all-cause NBP.

#### Case fatality rates

Case fatality rates for IPD published by Hoek et al. (2012) [16] were used. Since these data refer to different age groups and misses information on the distribution of deaths between meningitis and bacteremia, we assumed equal fatality rates for both indications. To be consistent with incidence estimations, data on case-fatality rates for inpatient NBP were based on Ewig et al. (2009) [17]. Both general mortality and case-fatality for acute cases of IPD and all-cause NBP depend upon age and risk profile.

### Clinical parameters

Table 2 presents all values used in the model for the baseline effectiveness (year 0) and the effectiveness over time.

**Table 2:** Effectiveness of PCV13 and PPSV23^a^.

Disease	Age Group	Risk Profile	PCV13, by No. of Years Since Receipt of Vaccine					PPSV23, by No. of Years Since Receipt of Vaccine					Source
			0	5	10	15	20	0	5	10	15	20	
**IPD** **(due to vaccine serotypes**^b^**)**	**18–49 yrs.**	LR/MR	85%	84%	60%	25%	0%	93%	77%	29%	4%	0%	PPSV23: [21–23] PCV13: [24, 25]
		HR	66%	66%	46%	20%	0%	21%	19%	9%	2%	0%	
	**50–59 yrs.**	LR/MR	82%	82%	56%	23%	0%	88%	71%	25%	3%	0%	
		HR	64%	64%	43%	18%	0%	15%	14%	37%	1%	0%	
	**60–64 yrs.**	LR/MR	80%	78%	49%	18%	0%	83%	63%	19%	2%	0%	
		HR	62%	61%	38%	14%	0%	8%	7%	3%	1%	0%	
	**65–74 yrs.**	LR/MR	77%	74%	1%	14%	0%	76%	54%	12%	1%	0%	
		HR	60%	58%	32%	11%	0%	2%	1%	0%	0%	0%	
	**75–99 yrs.**	LR/MR	70%	66%	24%	6%	0%	64%	34%	2%	0%	0%	
		HR	55%	52%	19%	4%	0%	0%	0%	0%	0%	0%	
**All-cause NBP**	**18–49 yrs.**	LR/MR	4%	4%	3%	1%	0%	0%	0%	0%	0%	0%	PPSV23: [21, 26–28] PCV13: [24, 25, 29]
		HR	3%	3%	2%	1%	0%	0%	0%	0%	0%	0%	
	**50–59 yrs.**	LR/MR	4%	4%	3%	1%	0%	0%	0%	0%	0%	0%	
		HR	3%	3%	2%	1%	0%	0%	0%	0%	0%	0%	
	**60–64 yrs.**	LR/MR	4%	4%	3%	1%	0%	0%	0%	0%	0%	0%	
		HR	3%	3%	2%	1%	0%	0%	0%	0%	0%	0%	
	**65–74 yrs.**	LR/MR	4%	4%	2%	1%	0%	0%	0%	0%	0%	0%	
		HR	3%	3%	1%	0%	0%	0%	0%	0%	0%	0%	
	**75–99 yrs.**	LR/MR	4%	3%	1%	0%	0%	0%	0%	0%	0%	0%	
		HR	2%	2%	1%	0%	0%	0%	0%	0%	0%	0%	

IPD = invasive pneumococcal disease | NBP = nonbacteremic pneumonia | LR = low-risk | MR = moderate-risk | HR = high-risk.

^a^ Revaccination was assumed to be equally effective as the initial dose.

^b^ Serotype coverage for PPSV23 ranges from 58.7–74.5%, and for PCV13, from 30.2–43.7% in year 1.

Note: Low-risk is specified as immunocompetent patients without any chronic medical conditions, moderate-risk describes immunocompetent patients with at least one chronic medical condition and high-risk represent immunocompromised/immunosuppressed patients, with or without chronic medical conditions (congenital or acquired).

#### Efficacy and effectiveness in year 1

**PPSV23:** Estimated values for vaccine-type IPD-effectiveness of initial vaccination with PPSV23 were derived from Smith et al. (2008) [23] (immunocompetent) and Shapiro et al. (1991) (immunocompromised) [22]. Effectiveness of PPV23 in the first year following receipt and subsequently among immunocompetent adults aged 18–49 years was assumed to be the same effectiveness for this age group than for the 50-year old group. For immunocompromised adults aged 18–49 years, initial effectiveness (21%) was based on data from Shapiro et al. (1991); for those aged 50–64 years, effectiveness was estimated by interpolating between values for persons aged 49 years (21%) and persons aged 99 years (0%) [21].

The PPSV23 effectiveness against all-cause NBP was assumed to be 0% based on various published sources and consistent with assumptions employed in published economic studies [21, 26–28]. This also refers to published meta-analyses, systematic reviews and base case analyses of other economic studies [23, 30–40]. A recently published meta-analysis [41] reported 64% effectiveness against pneumococcal NBP for PPSV23. To address this fact, we run a separate scenario (see section “Vaccination strategy”). Pletz et al. [42] report that 29.9% of all-cause NBP in Germany are caused by *S*. *pneumoniae*. Vaccine effectiveness from the meta-analysis therefore was combined with 29.9% [42] to gain vaccine effectiveness for all-cause NBP. resulting in a vaccine effectiveness of 19.1% against NBP (i.e., 64% x 29.9% = 19.1%). Effectiveness of PPV23 was assumed to be the same irrespective of prior vaccination experience with either PPV23 or PCV13 (i.e., potential vaccine hyporesponsiveness was not considered).

**PCV13:** For vaccination with PCV13 data for vaccine-type IPD for low/moderate-risk persons were based on the estimated effectiveness of PCV13 against vaccine-type IPD among subjects in the per-protocol population of CAPiTA [24]. For Germany we assumed that serotype coverage for PCV13 in IPD is 29.3% [43] for all age and risk groups. This serotype coverage was combined (i.e., multiplied) with vaccine effectiveness estimates to reduce effectiveness estimates such that they reflect estimates for IPD due to all serotypes. Estimated effectiveness (75.0%) against IPD was “anchored” to persons aged 73 years (mean age of study subjects in CAPiTA). Values were extrapolated to younger/older persons assuming age-specific differences for PCV13 were 50% of corresponding values for PPSV23. For high-risk adults, vaccine effectiveness is assumed to be 78% of corresponding values for the low/moderate-risk group based on a trial of pneumococcal vaccination in children with and without HIV [25]. Effectiveness of PCV13 was assumed to be the same irrespective of vaccination experience with either PPSV23 or PCV13. Effectiveness of each vaccine (i.e., PPSV23 and PCV13) across vaccine-specific serotypes was assumed to be the same. All of the above-noted assumptions are based on data, where available, and expert opinion, as needed.

Estimated effectiveness of PCV13 against vaccine-type NBP is presented by the value among subjects in the per-protocol population of CAPiTA (45%) [24]. At the time of modeling, there was no data available on serotype distribution within pneumococcal NBP for Germany so we assumed same distribution for NBP as for IPD (29.3%) [43] For the effectiveness against all-cause NBP, irrespective of setting of care, this value was multiplied with the percentage of all-cause NBP that was attributable to *S*. *pneumoniae* (29.9% in year 1 of modeling horizon) [42], resulting in 3.9% VE against all-cause NBP (45% x 29.9% 29.3% = 3.9% in year 1 of the modeling horizon). This value was “anchored” to persons aged 73 years (mean age of study subjects in CAPiTA) and was assumed to persist at this level for the initial 5 years of the modeling horizon for the reasons described above (data on file, Pfizer Inc.).

PCV13 effectiveness against NBP in high-risk adults was assumed to be 65% of corresponding values for low/moderate-risk persons [25]. Effectiveness estimates for high-risk persons employed in the model are consistent with data from French et al. on the effectiveness of PCV7 in HIV-infected adults [44]. Effectiveness of PCV13 was assumed to be the same irrespective of prior vaccination experience with either PPSV23 or PCV13.

#### Effectiveness over time

There are several publications in place describing a waning effectiveness of the polysaccharide vaccine over time [41, 45, 46]. To reflect this, we assumed that the effectiveness of PPSV23 in IPD wanes over time and from the first year on. We estimated rates of decay based on published literature. [21–23] The values represent percentage of decline in corresponding year from prior period. The decline in interval is estimated via exponential function.

Given the limited duration of follow-up in the trial populations we do also not assume lifelong protection from PCV13. Based on the literature and on the observation that vaccine effectiveness appears to be stable during the follow-up period (mean, 3.97 years) in CAPiTA, we assumed a constant protection of PCV13 over 5-year period [24, 47]. Because of increasing herd effects from year 1 to year 10 of the modeling horizon—and waning aside (see next section)—the “effective” effectiveness rates for PCV13 versus all-cause NBP was assumed to decline during this period. The rate of decline in PCV13 effectiveness over time after the initial 5-year period was assumed to be equal to 50% of PPSV23 values against IPD beginning with the first year following receipt of PPSV23. This assumption was changed to waning starting from the first year onwards in a sensitivity analysis (see section vaccination scenarios for details). For both vaccines the effectiveness was assumed to be 0% after year 16 of the model horizon. For more detailed information about derivation of vaccine effectiveness see S1 File.

#### Indirect effect (herd effect)

The introduction and use of the 7-valent pneumococcal conjugate vaccine (PCV7) among young children in the early 2000s has reduced the burden of IPD and pneumococcal NBP in this age group. It also resulted in reduced levels of IPD among older adults–which is discussed as the indirect effect [48–50]. For calculating the indirect effects (see Table 3), we used unpublished serotype-specific IPD data from National Reference Laboratory (NRZ) on Streptococcal Diseases [43] and the data for maximum herd effect for NBP reported by Pletz et al. (2016) [51].

**Table 3:** Maximum herd effect in year 5 of modelling (absolute in %).

	Age group (years)^a^					Source
	18–49 yrs.	50–59 yrs.	60–64 yrs.	65–74 yrs.	75–99 yrs.	
IPD	40.6	39.0	27.6	27.6	22.6	[43]
All-cause NBP (hospital.)	5.4	5.4	5.4	5.4	5.4	[51]
All-Cause NBP (outpatient)	5.4	5.4	5.4	5.4	5.4	

IPD = invasive pneumococcal disease | NBP = nonbacteremic pneumonia.

^a^ Effects are set equal for all risk groups.

To calculate the maximum herd effect for IPD we extrapolated the effects for the next 5 years (2016/17-2020/21) based on observed values from the past 5 years (2010/11-2015/16) for each age group [43]. The relationship between time and the distribution of different serotypes after vaccination was modelled using non-linear models. The performances and suitability of different functional forms were compared. Information criteria (Akaike AIC, Bayesian BIC) together with medical reasoning checking the plausibility of the model predictions were used to determine which function provided the most theoretically meaningful fit for each serotype (S1 Table). A percentage of the maximum reduction due to herd effect is realized in each of the first 5 years of the modeling horizon, with 100% of the reduction being reached in year 5 and carried forward through the end of the modelling horizon. The value from year 5 (default 100% of maximum reduction of incidence rate from year 1) is used in the calculation of the remaining years of the modelling horizon.

### Costs

#### Medical costs

Medical costs were calculated from inpatient and outpatient costs, according to national tariff manuals (see Table 4). Inpatient costs were based on data from the Institute for the Hospital Remuneration System (InEK) [52], where DRG specific calculations have been performed. For each ICD-10 code all associated DRGs were identified and a weighted average of DRG fees was calculated. All outpatient costs were based on physician costs (per case) and medication costs (with and without risk factors) based on duration and dosage [53–56]. We applied the official German Uniform Evaluation Scheme (EBM) [54] to calculate outpatient physician-related costs. For the medication costs we used the most often prescribed oral antibiotics for the outpatient treatment of NBP. Due to missing values for different risk groups we assumed the same costs for every risk group. All costs are presented in Euro for the year 2013.

**Table 4:** Age and risk level specific medical and non-medical costs^b^.

	Age/Risk Profile					
	18–49 yrs.	50–59 yrs.	60–64 yrs.	65–74 yrs.	75–99 yrs.	Source
	LR/MR/HR	LR/MR/HR	LR/MR/HR	LR/MR/HR	LR/MR/HR	
**Medical Costs (€)**						
*Costs for Hospitalization*						
Bacteremia	8,466	9,096	8,944	7,003	4,798	[52]
Meningitis	4,263	4,621	4,739	4,798	4,818	
All-cause NBP	2,981	3,615	3,848	3,600	3,029	
*Outpatient Costs*						
All-cause NBP	67	69	71	71	76	[53–56]^a^
**Non-medical Costs (€)**^b^						
*Work-loss Days*^c^						
Bacteremia	20.45	20.75	20.72	19.49	18.93	[52, 57, 58]^a^
Meningitis	16.78	16.69	16.65	16.64	16.63	
All-cause NBP (hospital.)	15.10	15.70	15.90	15.80	15.50	
All-cause NBP (outpatient)	14.00	14.00	14.00	14.00	14.00	
*Costs due to Productivity Loss*						
Bacteremia	1,775	1,784	1,014	144	26	[52, 57, 58] ^a^
Meningitis	1,457	1,434	815	123	23	
All-cause NBP (hospital.)	1,392	1,481	878	131	22	
All-cause NBP (outpatient)	1,392	1,481	878	131	22	

NBP = nonbacteremic pneumonia | LR = low-risk | MR = moderate-risk | HR = high-risk

^a^ Calculated values based on these references.

^b^ Not specified for risk group. Assumed equal relation between risk groups for all age-groups.

^c^ Only applied for employed persons.

Note: Low-risk is specified as immunocompetent patients without any chronic medical conditions, moderate-risk describes immunocompetent patients with at least one chronic medical condition and high-risk represent immunocompromised/immunosuppressed patients, with or without chronic medical conditions (congenital or acquired).

#### Non-medical costs

Non-medical costs represent the costs due to absence from work in consequence of the pneumococcal disease (see Table 4). The costs were calculated using the number of sick-leave days, the percentage of persons in the workforce (data of year 2012) and the value of a single sick-leave day (data of year 2014) based on the Human Capital Approach [52, 57]. We estimated the number of sick-leave days as the number of days according to the average length of stay in hospital plus seven additional calendar days of absence from work [58]. For outpatient care, we assume 14 sick-leave days per case based on an expert opinion. We were not able to differentiate non-medical costs by risk groups because of missing data.

#### Vaccination costs

To calculate the cost of vaccination we used the pharmacy retail price of a package size of ten for PCV13 and PPSV23 (62.93 € / 28.32 € per dose) [59], without any discounts. Administration costs were derived from a sample of German vaccination agreements between the Association of SHI Physicians and the SHI. Average reimbursement fee of 7.38 € for each injection was applied. Costs were tallied at model entry and at the time of revaccination. It was assumed that the vaccine is administered at the same time as other vaccines so no further visit costs are incurred.

### Utilities

The unit of effectiveness measure in our model are quality-adjusted life years (QALYs). The baseline utility weights are from a recently published analysis of Huber et al. (2016) [60] for utility in Germany (see Table 5). The reported values refer to the general population with and without underlying risk. Disutility values caused by pneumococcal disease were obtained from the study by Rubin et al. (2010) [61] who employed data from Melegaro (2004) [62].

**Table 5:** Age and risk level specific utilities.

	Age/Risk Profile										
	18–49 yrs.		50–59 yrs.		60–64 yrs.		65–74 yrs.		75–99 yrs.		Source
	LR	MR/HR	LR	MR/HR	LR	MR/HR	LR	MR/HR	LR	MR/HR	
**Utilities**	94.8	81.2	88.4	72.9	84.9	70.5	83.4	66.2	81.4	56.8	[60]^a^
**Reduction in Health-State Utility due to Disease**											
Bacteremia	0.0709										[61]^b^
Meningitis											
All-cause NBP (hospital.)											
All-cause NBP (outpatient)	0.0045										[62]^b^

NBP = community-acquired, non-bacteremic pneumonia | LR = low-risk | MR = moderate-risk | HR = high-risk

^a^ Calculated values based on these reference.

^b^ Not specified for risk group. Assumed equal relation between risk groups for all age-groups.

Note: Low-risk is specified as immunocompetent patients without any chronic medical conditions, moderate-risk describes immunocompetent patients with at least one chronic medical condition and high-risk represent immunocompromised/immunosuppressed patients, with or without chronic medical conditions (congenital or acquired).

### Vaccination strategy

The model compares the current vaccination strategy, recommended by the STIKO, with hypothetical strategies. For all of these scenarios, we assume a vaccination rate of 31.4% [2] with 5% (18-59y) respective 30% (≥60y) of the individuals being pre-vaccinated with PPSV23 within 6 years since the last receipt [2, 63]. The revaccination rate is assumed 100% where not mentioned separately (see Table 6).

**Table 6:** Overview of current STIKO recommendation and hypothetical strategies (#1 to#3).

	Percent vaccinated at model entry*							
	Current strategy		#1: sequential for all risk groups		#2: LR according to STIKO, sequential only for MR and HR		#3: LR and MR initial vaccination with PCV13, HR sequential	
Age Group	PPSV23	Seq	PPSV23	Seq	PPSV23	Seq	PCV13	Seq
18–59 yrs.								
LR	0	0	0	0	0	0	0	0
MR	31.4	0	0	31.4	0	31.4	31.4	0
HR	0	31.4	0	31.4	0	31.4	0	31.4
60–65 yrs.								
LR	31.4	0	0	31.4	31.4	0	31.4	0
MR	31.4	0	0	31.4	0	31.4	31.4	0
HR	0	31.4	0	31.4	0	31.4	0	31.4
66–99 yrs.								
LR	31.4	0	0	31.4	31.4	0	31.4	0
MR	31.4	0	0	31.4	0	31.4	31.4	0
HR	0	31.4	0	31.4	0	31.4	0	31.4

LR = low-risk | MR = moderate-risk | HR = high-risk

* Re-vaccination with PPSV23 every 6 yrs, no revaccination with PCV13

Seq = sequential vaccination: initial vaccination with PCV13, followed by PPSV23 after 6–12 month; re-vaccination with PPSV23 every 6 yrs with 100% revaccination rate.

Note: Low-risk is specified as immunocompetent patients without any chronic medical conditions, moderate-risk describes immunocompetent patients with at least one chronic medical condition and high-risk represent immunocompromised/immunosuppressed patients, with or without chronic medical conditions (congenital or acquired).

Overall, we evaluate seven scenarios. The first three vary the administration of sequential vaccination. All other scenarios are univariate sensitivity analysis of #1 (#4-#6) and #2 (#7) (see section “Sensitivity analysis”).

In scenario #1, we compare the current STIKO recommended strategy with a hypothetical strategy which assumes a sequential vaccination for all adults (except the low-risk <60y individuals). This scenario was chosen for practicability reasons at the vaccinating physician. Every person qualified for pneumococcal vaccination would get the same sequence of vaccination, with no differentiation. Physicians would not need to consider age and risk profile of the patient, making vaccination as easy as possible for the physician and therefore potentially increasing or at least keeping vaccination rates.

Scenario #2 assumes that the LR population for the age group 60 yrs. and older is vaccinated according to STIKO (PPSV23 at model entry and every 6 years) and the sequential strategy is provided for patients at increased risk for pneumococcal diseases (MR and HR group). This scenario was chosen for practical reasons since it appears to be difficult in daily routine to clearly differentiate medication-induced immuno-incompetent (e.g. treatment of rheumatoid arthritis) patients with moderate risk (polysaccharide vaccine only) from those with high risk (sequential vaccination). This scenario was chosen considering practicability reasons at the physician’s office as well as taking cost consideration into account. Differentiation between HR and MR might be difficult in cases, also patients currently falling into MR may qualify for HR in future. Not vaccinating LR patients sequentially on the other side saves one shot of PCV13 for the SHI.

A further scenario (#3) analyzes clinical impact and cost-effectiveness when PCV13 is initially administered for LR and MR (exchange of PPSV23 as initial strategy) and sequential only for HR. This scenario compares the current obligatory reimbursement strategy with a strategy substituting initial PPSV23 with PCV13. This strategy takes into consideration the better vaccination efficacy of PCV13 for all-cause NBP, as assumed in the base case model.

### Sensitivity and threshold analysis

#### Univariate sensitivity analysis

Scenarios #4 to #5 are one-way sensitivity analysis to #1. Some publications report a possible reduction of effectiveness over time for PCV13 [64, 65]. Hence, we assumed in #4 that PCV13 wanes immediately in #1 from the first year on.

While our base-case assumes no efficacy of PPSV23 to prevent pneumococcal NBP, STIKO refers to a systematic literature review and meta-analysis separately commissioned to and performed by the RKI. To address the findings of this recently published meta-analysis [41], we assumed a PPSV23 effectiveness of 64% for all-cause NBP in scenario #5.

Since the assumption of a revaccination rate of 100% might be too optimistic, we rerun #1 and #2 with a 50% revaccination rate as scenario #6 and #7.

#### Probabilistic sensitivity analysis

For each vaccination strategy, clinical outcomes and economic costs were simulated 1,000 times. These values were chosen to generate robust results and to ensure that the model runs given the computing power. We assume a log-normal distribution for costs and beta distributions for the epidemiological and effectiveness data. The alpha and beta were calculated from the standard deviations of actual data (see S3 Table). Expected life-time disease-related cases, deaths and costs in German adults are presented in detail in S4–S9 Tables including the mean differences between current and hypothetical vaccination strategy and corresponding 95% confidence intervals (CI).

Results of the Monte Carlo simulation for estimation of cost-effectiveness are shown in a scatterplot for each scenario with a willingness-to-pay threshold of €50,000. The scatter plot illustrates the uncertainty surrounding the estimates of expected incremental cost and expected incremental effect. The red line represents the willingness-to-pay thresholds of ICERs located to the right of the line in quadrant North-East are considered cost-effective.

## Results

### Outcomes

When running 1,000 simulations over a lifetime horizon, all scenarios showed a reduction of expected lifetime cases of IPD, pneumococcal NBP and death compared to the current scenario as recommended by STIKO. Most IPD cases were avoided in age group 18-59y and most NBP cases in age group >60y. This was seen for each scenario (see S4–S9 Tables).

The highest number of cases prevented was calculated in #1 (sequential vaccination for all risk groups and seniors): IPD -138 cases [95% CI -1,866; 1,392], NBP inpatient -43,000 cases [-57,000; -17,000], NBP outpatient -33,000 cases [-62,000; 3,000], -7,000 deaths [-15,000; 1,000], followed by #7 (vaccination of LR according to STIKO, MR/HR sequential strategy with 50% revaccination rate): IPD -110 cases [-1,854; 1,432], NBP inpatient -43,000 cases [-56,000; -17,000], NBP outpatient -30,000 cases [-58,000; 1,000], -7,000 deaths [-14,000; 1,000].

In contrast, the lowest number of preventable cases compared to the current strategy showed scenario #5. Assuming a 64% effectiveness NBP for PPSV23, implementing sequential vaccination would reduce IPD by -48 cases [-1,505; 1,075], NBP inpatient by -31,000 cases [-43,000; -18,000], NBP outpatient by -45,000 cases [-67,000; -26,000] and by -4,000 deaths [–10,000, 2,000] cases.

Estimated case reductions within scenario #3 (implementing the current strategy and initial vaccination with PCV13) are connected with large confidence intervals: IPD -72 cases [-13,284; 12,708], NBP inpatient -44,000 cases [-134,000; 47,000], NBP outpatient -33,000 cases [-142,000; 79,000], -6,000 deaths [-33,000; 21,000].

Savings for estimated lifetime costs of medical care were higher in all alternative scenarios as for non-medical and vaccination costs. Potential savings range between €-137 Mio. [-177 Mio.; 90 Mio.] (#1) and €-96 Mio. [-201 Mio.; 14 Mio.] (#5). Overall, all scenarios lead to extra costs. Highest difference in total costs was estimated in #5, when NBP effectiveness of PPSV23 was assumed to be 64%. The estimated upper and lower bound of the CI were closest in this scenario. Estimated difference in medical and vaccination costs was €481 Mio. [376 Mio.; 591 Mio.] and €462 Mio. [357 Mio.; 571 Mio.] if also including non-medical costs. On a patient level, the difference in costs is €7.13 [€5.57; €8.76] and €6.85 [€5.28; €8.47] respectively. Scenario #3 showed the lowest difference in total costs (medical and vaccination = €167 Mio. [-18 Mio.; 302 Mio.] / incl. non-medical €149 Mio. [-42 Mio.; 281 Mio.]), when vaccinating PCV13 first. Per patient, additional estimated costs were €2.48 [-0.27; 4.48] and 2.20 [-0.62; 4.16] respectively. This scenario resulted in the broadest estimated CI compared to the other scenarios.

### Cost-effectiveness

The cost-effectiveness results over all scenarios are shown in Table 7.

**Table 7:** Incremental cost-effectiveness ratios (ICER) over all scenarios (mean values).

Scenario	#1	#2	#3	#4	#5	#6	#7
Description	Sequential for all risk groups	LR according to STIKO, sequential only for MR and HR	LR and MR initial vaccination with PCV13, HR sequential	#1 with immediate waning for PCV13	#1 with 64% NBP Effectiveness PPSV23	#1 with 50% revaccination rate	#2 with 50% revaccination rate
*Payer Perspective*							
Cost per LYG	8,964	7,013	3,662	17,595	23,061	9,527	7,946
Cost per QALY gained	14,881	11,584	5,828	26,739	29,617	15,841	13,699
*Societal Perspective*							
Cost per LYG	8,447	6,459	3,258	15,084	22,146	8,960	7,290
Cost per QALY gained	14,023	10,667	5,186	22,923	28,441	14,898	12,570

NBP = community-acquired | non-bacteremic pneumonia | STIKO = German standing committee for vaccination | LYG = Life Year Gained | QALY = Quality Adjusted Life Year | LR = low-risk | MR = moderate-risk | HR = high-risk

Note: Low-risk is specified as immunocompetent patients without any chronic medical conditions, moderate-risk describes immunocompetent patients with at least one chronic medical condition and high-risk represent immunocompromised/immunosuppressed patients, with or without chronic medical conditions (congenital or acquired).

From the payer perspective and compared to all other scenarios, the incremental cost-effectiveness ratio (ICER) was the highest in #5 (€23,061 per LYG / 29,617 per quality-adjusted life year (QALY) gained), when assuming immediate waning of PCV13. The lowest ICER was predicted for #3 (€3,662 per LYG / 5,828 per QALY gained), when initial vaccination with PCV13 is assumed.

We estimate a lower ICER for sequential vaccination of the population with moderate and high-risk (#2) compared to sequential vaccination of all risk groups (#1), also when reducing the revaccination rate to 50% of #1 (#7).

### Threshold analysis

Table 8 presents the distribution of incremental costs and effects (LYG) represented by the scatterplots after 1,000 generated simulations which are included in the (see S1–S7 Figs).

**Table 8:** Proportion of simulations (%) in the quadrants of the ICER scatterplot.

Scenario	#1	#2	#3	#4	#5	#6	#7
Description	Sequential for all risk groups	LR according to STIKO, sequential only for MR and HR	LR and MR initial vaccination with PCV13, HR sequential	#1 with immediate waning for PCV13	#1 with 64% NBP Effectiveness PPSV23	#1 with 50% revaccination rate	#2 with 50% revaccination rate
SE: Dominant (hypothetical)	0	0	30	0	0	0	0
*NE*: *ICER >0 / ICER < WTP*	*66*	*66*	*28*	*60*	*62*	*64*	*65*
SW: ICER <0 / ICER < WTP	0	0	5	0	0	0	0
NE: ICER <0 and ICER > WTP	3	2	5	3	3	5	3
SW: ICER >0 and ICER > WTP	0	0	13	0	0	0	0
NW: Dominant (current)	31	32	20	37	35	31	33

STIKO = German standing committee for vaccination | ICER = Incremental Cost-Effectiveness Ratio | LR = low-risk | MR = moderate-risk | HR = high-risk | NW = North-West | NE = North-East | SW = South-West | SE = South-East | WTP = Willingness-To-Pay

Note: Low-risk is specified as immunocompetent patients without any chronic medical conditions, moderate-risk describes immunocompetent patients with at least one chronic medical condition and high-risk represent immunocompromised/immunosuppressed patients, with or without chronic medical conditions (congenital or acquired).

Quadrants description

SE–the hypothetical strategy is more effective and less costly compared to the current strategy.

NE–the hypothetical strategy is more effective but more costly compared to the current strategy.

SW–the hypothetical strategy is less effective but less costly compared to the current strategy.

NW–the hypothetical strategy is less effective and more costly compared to the current strategy.

In six of the seven scenarios, more than 60% of the ICERs predict cost-effective results for the hypothetical strategy from the payer perspective (as example see S1 Fig for #1).

Especially #3 reflects a high uncertainty of outcomes when implementing the hypothetical strategy and a wide range of ICER compared to the current strategy. 63% of the ICER favors initial vaccination of PCV13 (hypothetical strategy), 30% of them dominant.

## Discussion

In our study, we evaluated the cost-effectiveness of the newly implemented pneumococcal vaccination scheme in Germany compared to other, hypothetical strategies that could serve as voluntary services for sick funds. As far as we know, this is the first evaluation of the current setting addressing this question. Since the German healthcare system allows statutory health insurances to differentiate themselves by offering additional voluntary services to their insured [6] on top of obligatory services, we modelled a variety of different vaccination strategies in Germany, which can be offered as additional voluntary services for pneumococcal vaccination and also prevent more cases. Based on the scenarios discussed in this paper, our results predict that all hypothetical strategies are mostly cost-effective compared to the existing policy, despite the wide range of confidence intervals. In all scenarios, additional cases of IPD, NBP and deaths could be avoided. The avoided number of cases was the highest when vaccinating all risk groups with the sequential strategy (#1), even though the ICER of #2 and #7 (LR according to STIKO, sequential only for MR and HR) was lower than for #1. That consequently leads to savings in medical and non-medical costs. Depending on the scenario, these savings ranged from €136 Mio. (#3, LR and MR initial vaccination with PCV13, HR sequential) to €187 Mio (#4: #1 with immediate waning for PCV13) on the population level. Due to the expanded use of PCV13, the lifetime vaccination costs increased compared to the current, recommended strategy (from €284 Mio. in #3 to €577 Mio. in #1, #4, #5, #6). In total, additional costs per patient ranged from €2.48 - €7.13 for the payer and €2.20 – €6.85 for the society.

Hence, our model disclosed that within each scenario every case avoided has its own price. However, in more than two-third of the hypothetical scenarios the ICERs were below the present maximum WTP of €50,000. Although there is no clear-cut margin in the German health care system, a cost-effectiveness threshold of $50,000 for a life year or QALY gained is assumed internationally [66]. With respect to our study, results seem well below the boundaries of societies’ and payers’ WTP [66, 67].

The main strength of our study is that we compare different vaccination strategies (not only two single vaccines) for the entire target population of the recommendation and capture the underlying uncertainty of each scenario within a microsimulation approach. While the health economic model as basis for the current German pneumococcal recommendation evaluates various vaccination strategies for seniors [2], the model presented here takes into consideration all individuals (i.e. healthy seniors as well as patient with varying underlying risks for pneumococcal infections). Our results therefore give an optimal basis for an informed decision-making process.

We were able to identify German data for most of the input factors, but uncertainty still exists regarding the extent of vaccination in Germany. We did not find reliable data for the vaccination rate nor for the rate of revaccination. Assumption has to be made what influences the results for both strategies in the scenarios. Theidel et al. (2013) [63] tried to predict the vaccination rate for Germany of the adult pneumococcal vaccination based on claims data. One of the main limitations was the short study period of a total of four quarters for the claims data. Therefore, we decided to use the data of a population-based survey [5]. The survey evaluates the vaccination status in Germany. However, information is limited to the vaccination rate of the overall population without differentiating risk-groups. Further, the survey asked for at least one dose of pneumococcal vaccination, so that no information was given regarding revaccination rates.

No data on disease incidence are available for Germany. Our model uses incidence estimates from Reinert et al. (2005) [15] for IPD and Schnoor et al. (2007) [19] for NBP, since these were the only available estimates at the time of model development. Newer estimates on IPD were published by the RKI in the rationale of the decision on recommendation [2]. The RKI refers to cases reported as main diagnosis in the German hospital statistics (Krankenhaus-Diagnosestatistik, year 2010–2013) and therefore is based on a passive reporting system, whereas Reinert et al. (2005) [15] base their estimates on an active surveillance in the most populated federal state of Germany. Besides the difference in reporting system, it is very likely that the ICD-10-codes as identified as IPD within the hospital statistics report the more severe cases only, whereas Reinert et al. (2005) [15] detects all cases within the active surveillance system. Moreover, besides the differences in the incidence estimates in both references, detection of IPD depends on a positive culture. However, cultures of blood or liquor are not always part of routine diagnostics and patients pre-treated with antibiotics will no more show positive culture. Taking all this into consideration, we believe the incidence estimate used in our model is close to reality. Regarding estimated incidence on NBP, Schnoor et al presented several approaches for estimation of incidence, with 8.7/1000 being valued as the most reliable. This was used for our model. Combining these values with data from Federal Health Monitoring [14] resulted in a estimated hospitalization rate of 42%. Kolditz et al. (2016) [68] derived very similar results for all-cause NBP in their retrospective cohort study based on statutory health insurance data in the German federal state of Saxony. Assuming approximately 20% of NBP [2] caused by *S*. *pneumonia*, these estimates also are in line with our assumptions in the model.

Newer data show that the rate for senior people affected by hospitalized NBP increases over time, whereas the rate for younger people decreases [69]. As a consequence, for our model calculation, this circumstance might positively influence the impact of the adult pneumococcal vaccination, despite of the indirect effects from children vaccination. Pelton et al. (2015) [20] reported for NBP, that incidences for LR and HR increases over time, MR decreases. Assuming that the risk profile for pneumococcal vaccination might be balanced, it would not influence the cost-effectiveness results of our model.

We also had made assumptions regarding costs per risk group. Since no risk specific data exist, our model considers only the cost by population size per age group. Also, remuneration schemes do not reflect the real cost of a disease. They only report the reimbursed cost by the SHI, what is sufficient when reporting results from the payer perspective but not from the social perspective. The human capital approach used for estimation of indirect costs assumes that every sick person causes costs in the social system because no economic and social return will yield. In contrast, the friction cost approach would assume that a sick person might be replaced by an unemployed, what would relativize the resulting system costs. Our estimations on days of sick-leave for inpatient cases are based on general data from federal statistics and not risk, age or disease specific. For outpatient cases we have to refer to an expert opinion what is also of limited evidence.

The utility figures used for our model are a more conservative approach. The CAPiTA piggyback study [70] shows greater decrements, which would improve the ICER due to the positive effect of PCV13 on NBP. Also, the mortality rate for outpatient NBP was chosen conservative. A recently published study reports an outpatient mortality rate of 5% which was strongly age dependent and fell to 0.3% for patients with age <60 years [68]. Non-survivors had a median age of 86 and high comorbidity rates.

Uncertainty also refers to the assumed effectiveness of vaccination including the effectiveness over time. Due to the lack of data we applied simplifying assumptions, which in some cases might not be realistic. Most data come from clinical trials which are likely to not distinguish different risk groups. Also, the comparability of the trial populations, settings and endpoints is limited. To our knowledge, there is no head-to-head clinical study which compared PPSV23 with PCV13. For IPD, we used data of a cost-effectiveness study looking at PPSV23 [23] and efficacy data from Shapiro et al. (1991) [22]. The cost-effectiveness study is one of the few studies which show IPD effectiveness by age (including those <65y and <55y) and time since vaccination. These estimates are based on a Delphi panel of experts, which relied primarily on efficacy data from Shapiro et al. (1991). NBP contributes the majority to the total burden of disease. Whereas the value of PPSV23 to prevent IPD is well recognized, published data for PPSV23 effectiveness in NBP is still subject of controversy.[71] To be consistent, we refer to assumptions and data employed in other clinical and economic studies [23, 30–40]. Due to computational resources and the duration of each simulation, we had to reduce ourselves to only simulate extreme cases: To address the recently published results of a meta-analysis we assumed an NBP protective effect of 64% [41] against any pneumococcal pneumonia for PPSV23 in #5 instead of assuming no protective effect for #1.

Further, there was no reliable information on the duration of the effectiveness over time, neither for PPSV23 nor for PCV13. Based on a recently published trial [24], we assumed declining effectiveness for PCV13 in all scenarios from the fifth year onwards in the base case. This assumption affects the impact of PCV13 positively. Therefore, we integrated assumptions based on data for PPSV23 and assumed waning for PCV13 (#4) from the first year on.

Another aspect influencing the effectiveness is replacement of vaccine-type serotypes by serotypes not covered by a vaccine. Therefore, and to predict indirect effects, we used data from the NRZ [43], to simulate the serotype-coverage for IPD for the past and future five-year time horizon. This approach leads to a situation, that after reaching the steady-state induced by children vaccination, some PCV13-serotypes will remain. Other models predict that all but one PCV13 serotypes will eradicate completely [72]. Personal communication with experts [73] confirmed that this is most unlikely and also other serotypes will remain.

Caused by the lack of reliable data for all age groups and simplification purposes, effectiveness of PCV13 and PPSV23 was assumed to be the same irrespective of prior vaccination experience with either PPSV23 or PCV13. There is limited evidence about the clinical impact on immune response of PVC13 when initial vaccination with PPSV23. Greenberg et al. (2014) found that subjects who received PPSV23 as the initial study dose had lower opsonophagocytic activity (OPA) antibody responses after subsequent administration of PCV13 than those who had received PCV13 as the initial dose followed by a dose of PPSV23. This effect remained regardless of the level of the initial OPA response to PPSV23 [46]. A review by Falkenhorst et al. [41] describes timely limitations of hypo-responsiveness for PPSV23, what also justifies our assumption. Clinical consequences and relevance has not been further studied for both vaccines.

The difference between other health economic models evaluating the pneumococcal vaccination for Germany [35, 72, 74] are not only the data used for costs and effectiveness but also the evaluated strategies (past and current). Two of them [35, 74] evaluate the cost-effectiveness using static Markov models, without illustrating the current sequential vaccination programs. The most recently published [72] shows first results of evaluating a sequential strategy in elderly. This is also the only model that reflects the transmission dynamic by using differential equations. Despite differences in model structure, model population, methods of model estimation, and vaccination strategies considered, the conclusions that may be drawn from this study were produced using a model that is consistent with those from recent cost-effectiveness analyses of alternative adult vaccination strategies against pneumococcal disease [26].

Due to uncertain input parameters, all models are subjects to restrictions and show variability in results, especially those with transmission dynamics [75]. Nevertheless, the positive impact of indirect effects of children vaccination with PCV13 needs to be recognized, even if the models do not reflect the cost-effectiveness of the entire new STIKO recommendation since it neglects differentiation between risk groups [2].

Modelling a health decision problem requires a balance between the necessary number of assumptions and an adequate level of uncertainty. The comparison of results between models should take into account the heterogeneity of the various modelling approaches. No single type of model can provide adequate answers under all circumstances.

In the light of growing budgetary pressures, there is a rising demand for comprehensive evaluations of the impact of current vaccination strategies and hypothetical alternatives, which requires microsimulation.[7] The chosen microsimulation framework with Markov-type process allows comparing different vaccination scenarios of sequential vaccination (current vs. hypothetical). A simple Markov model would not be able to produce similar results [76]. Also, this type of modelling helps to explore uncertainty around model outcomes in estimating ranges. It has the flexibility to analyze a wide variety of scenarios, but results vary from run to run because of the stochastic nature. The simulation’s main limitation is the length of time required to run a given scenario. The stochastic nature of the model requires to run multiple simulations for a given analysis in order to properly converge on the expected result. Furthermore, in sequential decision models, parameter uncertainty may have a higher impact on the confidence in model outcomes than it would in one-time decision models.

Compared to a dynamic model approach, our model contains less assumptions regarding uncertain parameters e.g. the case-carrier-ratio. But it does not fully reflect the population dynamics which might be necessary in the presence of interactions between individuals [77]. To examine the long-term effects of a vaccination strategy it would be desirable to include an algorithm to model the transmission dynamic of the pneumococcal carriage and pneumococcal disease.[75] Nevertheless in such models, the model calibration and the probabilistic sensitivity analysis become more challenging and computationally intensive because of the uncertainty of data and variability of results. The recently published report [72] shows how challenging it is, especially to find reliable data for Germany.

While the consideration of cost-effectiveness is an important component in the evaluation of vaccines and vaccination strategies, the use of cost-effectiveness models can be problematic if used as an absolute criterion. This is because of the uncertain nature of models which necessarily include incomplete data and incorrect assumptions prior to vaccine introduction. Routinely used it runs the risk of transforming vaccines into a tool for achieving cost savings within the health care system rather than a public health intervention targeting human suffering, death and disability. Cost-effectiveness analysis should be a standard tool for health care decisions but not as a single criterion.[78] If the primary goal is to prevent disease morbidity and mortality, cost-effectiveness analyses should play a secondary role in the vaccine decision-making processes [79]. In this case, cost-effectiveness analyses should rather be used as a “gating criterion” to decide which vaccines should be developed or which strategy should be implemented.

## Conclusion

The newly recommended sequential strategy for pneumococcal vaccination of immunocompromised adults in Germany improves immunization for patients with high risk of pneumococcal disease. Our study indicates that a sequential vaccination strategy independent of underlying risks might be optimal in minimizing the burden of pneumococcal disease. All hypothetical strategies would lower the number of pneumococcal disease cases and deaths compared to the current STIKO recommendation and vaccination directive. Some of the hypothetical strategies might also be easier to implement in daily routine. All hypothetical strategies seem to be a reasonable choice in terms of cost-effectiveness. Further research on the public health and economic impact is needed to confirm our results in a real-world setting.

## Supporting information

S1 File Derivation of vaccine effectiveness. (PDF)

S1 Table Model fits for serotype distribution IPD over time (incl. prognosis for next 5 years). (PDF)

S2 Table Development of herd effect by year of modelling (cumulative in %) based on ST-specific IPD data. (PDF)

S3 Table Distribution of PSA parameters. (PDF)

S4 Table Expected lifetime disease-related cases, deaths, and costs in German adults #1-#4. (PDF)

S5 Table Expected lifetime disease-related cases, deaths, and costs in German adults #5-#7. (PDF)

S6 Table Expected lifetime disease-related cases, deaths, and costs in German adults <60 years (#1-#4). (PDF)

S7 Table Expected lifetime disease-related cases, deaths, and costs in German adults <60 years (#5-#8). (PDF)

S8 Table Expected lifetime disease-related cases, deaths, and costs in German adults ≥60 years (#1-#4). (PDF)

S9 Table Expected lifetime disease-related cases, deaths, and costs in German adults ≥60 years (#5-#7). (PDF)

S1 Fig ICER scatterplot for #1. (TIF)

S2 Fig ICER scatterplot for #2. (TIF)

S3 Fig ICER scatterplot for #3. (TIF)

S4 Fig ICER scatterplot for #4. (TIF)

S5 Fig ICER scatterplot for #5. (TIF)

S6 Fig ICER scatterplot for #6. (TIF)

S7 Fig ICER scatterplot for #7. (TIF)
