# Supplementary material for: Estimating the cost-effectiveness of a sequential pneumococcal vaccination program for adults in Germany
Source: PLoS One. 2018 May 24;13(5):e0197905. doi: 10.1371/journal.pone.0197905 (PMC5967715; doi:10.1371/journal.pone.0197905)
Supplement: S1 File — (PDF) [file pone.0197905.s001.pdf]

S1 File. Derivation of Vaccine Effectiveness

Vaccine Effectiveness of PPSV23 against VT-IPD

Vaccine effectiveness (VE) of vaccination with PPSV23 against VT (vaccine type)-IPD—by age and time since vaccination—among low- and high-risk adults aged ≥50 years, and immunocompromised adults aged ≥65 years, was based on published data from Smith et al. 2008 [1]:

Table 2 Expert panel estimates of PPV effectiveness (%) in preventing vaccine-susceptible invasive pneumococcal disease

Years post vaccination	Vaccinee age/health								
	Healthy 50-year-olds[a]			Healthy 65-year-olds[a]			Healthy 80-year-olds[a]		
	Base case	Range		Base case	Range		Base case	Range	
		Low	High		Low	High		Low	High
1	93	80	95	80	60	90	67	20	85
3	89	74	94.5	73	50	83	53	0	83.5
5	85	66	90	58	30.5	80	32	0	75
7	60	40	75	33	13	48	10	0	30
10	20	0	30	0	0	10	0	0	10
15	0	0	20	0	0	10	0	0	10

[a] Patients with other comorbid conditions vaccinated at these ages had the same base case and high range values, low range was decrease

Estimates reported by Smith and colleagues were based on a Delphi panel of experts, which relied primarily on data from Shapiro et al. 1991 [2]:

Table 5. Aggregate Protective Efficacy of Polyvalent Pneumococcal Vaccine against Serotypes in the Vaccine in Immunocompetent Patients, According to Age Group and Time since Vaccination.

Age (yr)	No. of Case-Control Pairs	Time since Vaccination*		
		<3 yr	3–5 yr	>5 yr
		percent protective efficacy† (95 percent confidence interval)		
<55	125	93 (82 to 97)	89 (74 to 96)	85 (62 to 94)
55–64	149	88 (70 to 95)	82 (57 to 93)	75 (38 to 90)
65–74	213	80 (51 to 92)	71 (30 to 88)	58 (−2 to 83)
75–84	188	67 (20 to 87)	53 (−15 to 81)	32 (−67 to 72)
≥85	133	46 (−31 to 78)	22 (−90 to 68)	−13 (−174 to 54)

VE Matrix

For persons aged 50, 65, and 80 years, vaccine effectiveness was estimated to be the values as set forth in Smith et al. (2008) [1]:

Table. Vaccine effectiveness of PPSV23 against vaccine-type IPD

	Years From Vaccination						
**Age**	**0**	**1**	**2**	**3**	**4**	**5**	**...**
...							
48							
49							
**50**	**93.00**						
51							
52							
...							
**65**	**80.00**						
...							
**80**	**67.00**						
...							

Effectiveness of PPSV23 in the first year following receipt and subsequently among immunocompetent adults aged 18-49 years was assumed to be the same as that for persons aged 50 years:

Table. Vaccine effectiveness of PPSV23 against vaccine-type IPD

	Years From Vaccination						
**Age**	**0**	**1**	**2**	**3**	**4**	**5**	**...**
**...**	**93.00**						
**48**	**93.00**						
**49**	**93.00**						
50	93.00						
51							
52							
...							
65	80.00						
...							
80	67.00						
...							

For those aged 51-64 years and 66-99 years, effectiveness was linearly interpolated using the values for persons aged 50 years (93%) and 60 years (80%), and 60 years (80%) and 80 years (67%), respectively, in 1-year increments:

Table. Vaccine effectiveness of PPSV23 against vaccine-type IPD

	Years From Vaccination						
**Age**	**0**	**1**	**2**	**3**	**4**	**5**	**...**
...	93.00						
48	93.00						
49	93.00						
50	93.00						
**51**	**92.13**						
**52**	**91.27**						
...							
**64**	**80.87**						
65	80.00						
**66**	**79.13**						
...	...						
**79**	**67.87**						
80	67.00						
**81**	**66.13**						
...	...						

The assumed rate of decline in protection by time since vaccination was also based on data from Smith et al. 2008 [1]:

Table. Vaccine effectiveness of PPSV23 against vaccine-type IPD

	Years From Vaccination								
**Age**	**0**	**1**	**2**	**3**	**4**	**5**	**6**	**7**	**...**
...	93.00								
48	93.00								
49	93.00								
50	93.00				**89.00**		**85.00**		
51	92.13								
52	91.27								
...	...								
64	80.87								
65	80.00								
66	79.13								
...	...								
79	67.87								
80	67.00				**53.00**		**32.00**		
81	66.13								
...	...								

Values were then linearly interpolated based on the Smith data points, by age:

Table. Vaccine effectiveness of PPSV23 against vaccine-type IPD

	Years From Vaccination								
**Age**	**0**	**1**	**2**	**3**	**4**	**5**	**6**	**7**	**...**
...	93.00				**89.00**		**85.00**		
48	93.00				**89.00**		**85.00**		
49	93.00				**89.00**		**85.00**		
50	93.00				89.00		85.00		
51	92.13				**87.80**		**83.23**		
52	91.27				**86.60**		**81.47**		
...	...				**...**		**...**		
64	80.87				**72.20**		**60.27**		
65	80.00				**71.00**		**58.50**		
66	79.13				**69.80**		**56.73**		
...	...				**...**		**...**		
79	67.87				**54.20**		**33.77**		
80	67.00				53.00		32.00		
81	66.13				**51.80**		**30.23**		
...	...				**...**		**...**		

And then by years since receipt:

Table. Vaccine effectiveness of PPSV23 against vaccine-type IPD

	Years From Vaccination								
**Age**	**0**	**1**	**2**	**3**	**4**	**5**	**6**	**7**	**...**
...	93.00	92.00	91.00	90.00	89.00	87.00	85.00	83.00	...
48	93.00	92.00	91.00	90.00	89.00	87.00	85.00	83.00	...
49	93.00	92.00	91.00	90.00	89.00	87.00	85.00	83.00	...
50	93.00	92.00	91.00	90.00	89.00	87.00	85.00	83.00	...
51	92.13	91.05	89.97	88.88	87.80	85.52	83.23	80.95	...
52	91.27	90.10	88.93	87.77	86.60	84.03	81.47	78.90	...
...	...	...	...	...	...	...	...	...	...
64	80.87	78.70	76.53	74.37	72.20	66.23	60.27	54.30	...
65	80.00	77.75	75.50	73.25	71.00	64.75	58.50	52.25	...
66	79.13	76.80	74.47	72.13	69.80	63.27	56.73	50.20	...
...	...	...	...	...	...	...	...	...	...
79	67.87	64.45	61.03	57.62	54.20	43.98	33.77	23.55	...
80	67.00	63.50	60.00	56.50	53.00	42.50	32.00	21.50	...
81	66.13	62.55	58.97	55.38	51.80	41.02	30.23	19.45	...
...	...	...	...	...	...	...	...	...	...

For immunocompromised persons aged 50 years, vaccine effectiveness against VT-IPD was estimated using data from Shapiro et al. (1991) [2]:

Table 4. Aggregate Protective Efficacy of Polyvalent Pneumococcal Vaccine against Serotypes in the Vaccine.*

Group†	No. of Patients	Percent Vaccinated‡		Discordant Pairs§	Matched Odds Ratio	Protective Efficacy (95% Confidence Interval)	χ^2	P Value
		case patients	controls			*percent*		
All patients	983	12.5	19.8	66/151	0.44	56 (42 to 67)	33.3	<0.00001
Stratum 1	175	22.3	22.3	15/19	0.79	21 (−[illegible]5 to 60)	0.5	0.48
Stratum 2	808	10.4	19.3	51/132	0.39	61 (47 to 72)	35.9	<0.00001

And for immunocompromised persons aged 69 years, Fry et al. (2002) [3]:

Vaccines and assumptions	Base case
23-valent polysaccharide vaccine	
Maximum VE against IPD in healthy	
65-year-old	75%
75-year-old	60%
85-year-old	34%
VE against IPD, immune compromised persons	0%
VE against non-bacteremic pneumococcal pneumonia	0%

VE Matrix

Table. Vaccine effectiveness of PPSV23 against vaccine-type IPD (high-risk)

	Years From Vaccination						
**Age**	**0**	**1**	**2**	**3**	**4**	**5**	**...**
...							
48							
49							
**50**	**21.0**						
51							
52							
...							
**69**	**0.0**						
...							

As with immunocompetent persons aged 18-49, VE was assumed to be equal to that of a 50-year-old:

Table. Vaccine effectiveness of PPSV23 against vaccine-type IPD (high-risk)

	Years From Vaccination						
**Age**	**0**	**1**	**2**	**3**	**4**	**5**	**...**
**...**	**21.0**						
**48**	**21.0**						
**49**	**21.0**						
50	21.0						
51							
52							
...							
69	0.0						
...							

For persons aged 51-68, values were extrapolated by interpolating between the two data points (i.e., 21% for 50-year olds and 0% for 69 year olds). For persons aged >69 years, effectiveness was assumed to be 0%:

Table. Vaccine effectiveness of PPSV23 against vaccine-type IPD (high-risk)

	Years From Vaccination						
**Age**	**0**	**1**	**2**	**3**	**4**	**5**	**...**
...	21.00						
48	21.00						
49	21.00						
50	21.00						
**51**	**19.89**						
**52**	**18.79**						
...	...						
69	0.00						
...	**0.00**						

Vaccine Effectiveness of PCV13 against IPD

VE-PCV13 against VT-IPD was estimated using data from CAPiTA [4].

Table 4. Vaccine Efficacy for the Secondary Efficacy Endpoint of First Episode of VT-IPD – Per-Protocol Case-Level Population

		Vaccine Group				
		13vPnC N[a]=42240	Placebo N[a]=42256			
Efficacy Endpoint	Total Number of Episodes	n[b]	n[b]	VE	(95% CI[c])	p-Value[d]
First episode of VT-IPD	35	7	28	75.00	41.43, 90.78)	0.0005

Note: The SSUAD assay does not distinguish between the vaccine-type serotype and closely related serotype(s) within their respective serogroup for 6A and 6C; 7F and 7A; 9V and 9A; 18C and 18A, 18B, and 18F.
a. N = number of subjects who received the vaccine in the vaccine group.
b. n = Number of subjects who experienced a first case of the specific event during the study.
c. Confidence intervals were derived using the Clopper-Pearson method. The lower limit of this confidence interval must exceed 0.0 to conclude efficacy for this final analysis.
d. p-Value for the null hypothesis that VE=0.
Program ID: Study 6115A1-3006-WW-NL/CP EFC_SCND.SAS. Runtime ID: 11FEB2014 01:00

The mean age from CAPiTA [4] was 73 years. Thus, the 75% effectiveness estimate from the table above was “anchored” around persons aged 73 years:

Table. Vaccine effectiveness of PCV13 against vaccine-type IPD

	Years From Vaccination						
**Age**	**0**	**1**	**2**	**3**	**4**	**5**	**...**
...							
71							
72							
**73**	75.00						
74							
75							
...							

VE-PCV13 was assumed to be constant (i.e., durable) over 5-year period (ie, through year 4), based on mean duration of follow-up in CAPiTA [4]:

Table. Vaccine effectiveness of PCV13 against vaccine-type IPD

	Years From Vaccination						
**Age**	**0**	**1**	**2**	**3**	**4**	**5**	**...**
...							
71							
72							
**73**	75.00	75.00	75.00	75.00	75.00		
74							
75							
...							

Values were extrapolated to younger/older persons assuming age-specific differences for PCV13 were 50% of corresponding values for PPSV23:

PPSV23

	Years Since Vaccination					
Age	0	1	2	3	4	5
...						
70	75.7					
71	74.8					
72	73.9					
73	73.1					
74	72.2					
75	71.3					
...						

PCV13

	Years Since Vaccination					
Age	0	1	2	3	4	5
...						
70	76.3					
71	75.9					
72	75.4					
73	75.0	75.0	75.0	75.0	75.0	
74	74.6					
75	74.1					
...						

For each age, VE-PCV13 was assumed to be durable:

PPSV23

	Years Since Vaccination					
Age	0	1	2	3	4	5
...						
70	75.7					
71	74.8					
72	73.9					
73	73.1					
74	72.2					
75	71.3					
...						

PCV13

	Years Since Vaccination					
Age	0	1	2	3	4	5
...						
70	76.3	76.3	76.3	76.3	76.3	
71	75.9	75.9	75.9	75.9	75.9	
72	75.4	75.4	75.4	75.4	75.4	
73	75.0	75.0	75.0	75.0	75.0	
74	74.6	74.6	74.6	74.6	74.6	
75	74.1	74.1	74.1	74.1	74.1	
...						

To extrapolate age-specific values beyond 5th-year of modeling horizon, 50% of observed decrease in VE-PPSV23 between respective 1-year time increments was applied to PCV13 values (staggered by 5-year assumed durability):

PPSV23

	Years Since Vaccination					
Age	0	1	2	3	4	5
...						
70	75.7	71.1				
71	74.8	70.0				
72	73.9	68.9				
73	73.1	67.8				
74	72.2	66.7				
75	71.3	65. 6				
...						

PCV13

	Years Since Vaccination					
Age	0	1	2	3	4	5
...						
70	76.3	76.3	76.3	76.3	76.3	74.0
71	75.9	75.9	75.9	75.9	75.9	73.5
72	75.4	75.4	75.4	75.4	75.4	72.9
73	75.0	75.0	75.0	75.0	75.0	72.3
74	74.6	74.6	74.6	74.6	74.6	71.7
75	74.1	74.1	74.1	74.1	74.1	71.1
...						

VE-PCV13 is assumed to be 0 by—at latest—year 16 of modeling horizon. For high-risk persons, VE-PCV13 was assumed to be 78% of corresponding values for low/moderate-risk persons based on data from Klugman et al. (2003) [5] (65% versus 83%):

Table 2. First Episodes of Invasive Pneumococcal Disease.*

Variable	Vaccinated Group	Control Group	P Value	Vaccine Efficacy (95% CI)
	no. of episodes			*percent*
HIV-negative children				
Invasive pneumococcal disease	11	19	0.2	42 (−28 to 75)
Vaccine-serotype pneumococci	3	17	0.003	83 (39 to 97)
Non–vaccine-serotype pneumococci	4	1	0.38	−300 (−19,599 to 60)
Vaccine-related–serotype pneumococci	4	1	0.38	−300 (−19,599 to 60)
HIV-positive children				
Invasive pneumococcal disease	22	47	0.004	53 (21 to 73)
Vaccine-serotype pneumococci	9	26	0.006	65 (24 to 86)
Non–vaccine-serotype pneumococci	9	8	1	−13 (−235 to 62)
Vaccine-related–serotype pneumococci	6	16	0.05	63 (−1 to 88)
All children				
Invasive pneumococcal disease	33	66	0.001	50 (23 to 68)
Vaccine-serotype pneumococci	12	43	<0.001	72 (46 to 87)
Non–vaccine-serotype pneumococci	13	9	0.52	−44 (−283 to 43)
Vaccine-related–serotype pneumococci	10	17	0.25	41 (−36 to 75)

* Vaccine-serotype pneumococci were serotypes 1, 4, 5, 6B, 9V, 14, 18C, 19F, and 23F. Vaccine-related pneumococci were serotypes 6A, 19A, and 19B. CI denotes confidence interval, and HIV human immunodeficiency virus. For HIV-positive children and for all children, the sum of episodes involving vaccine, nonvaccine, and vaccine-related serotypes exceeds the number of episodes of invasive pneumococcal disease because only the first episode of invasive disease was counted.

Vaccine Effectiveness for PPSV23 against VT-NBP

VE-PPSV23 against VT-NBP was assumed to be 0% [3, 6-8].

Vaccine Effectiveness for PCV13 against VT-NBP

VE-PCV13 against VT-NBP was taken from CAPiTA [4]:

Table 3. Vaccine Efficacy for the Secondary Efficacy Endpoint of First Episode of Confirmed NB/NI VT Pneumococcal CAP – Per-Protocol Case-Level Population

		Vaccine Group				
		13vPnC N[a]=42240	Placebo N[a]=42256			
Efficacy Endpoint	Total Number of Episodes	n[b]	n[b]	VE (%)	(95.2% CI[c])	p-Value[d]
First episode of confirmed NB/NI VT pneumococcal CAP	93	33	60	45.00	(14.21, 65.31)	0.0067

Note: The SSUAD assay does not distinguish between the vaccine-type serotype and closely related serotype(s) within their respective serogroup for 6A and 6C; 7F and 7A; 9V and 9A; 18C and 18A, 18B, and 18F.

a. N = number of subjects who received the vaccine in the vaccine group.

b. n = Number of subjects who experienced a first case of the specific event during the study.

c. Confidence intervals were derived using the Clopper-Pearson method. The lower limit of this confidence interval must exceed 0.0 to conclude efficacy for this final analysis.

d. p-Value for the null hypothesis that VE=0.

Program ID: Study 6115A1-3006-WW-NL/CP EFC_SCND.SAS. Runtime ID: 11FEB2014 01:00

As with VE-PCV13 against VT-IPD, this estimate (45%) was anchored around the mean age of the trial (i.e., persons aged 73 years):

Table. Vaccine effectiveness of PCV13 against vaccine-type NBP

	Years From Vaccination						
**Age**	**0**	**1**	**2**	**3**	**4**	**5**	**...**
...							
71							
72							
**73**	**45.00**						
74							
75							
...							

VE-PCV13 was assumed to be constant (i.e., durable) over 5-year period (ie, through year 4), based on mean duration of follow-up in CAPiTA [4]:

Table. Vaccine effectiveness of PCV13 against vaccine-type NBP

Age	Years From Vaccination						
	0	1	2	3	4	5	...
...							
71							
72							
**73**	45.00	**45.00**	**45.00**	**45.00**	**45.00**		
74							
75							
...							

Values were extrapolated to younger/older persons assuming age-specific differences for PCV13 were 50% of corresponding values for PPSV23 against IPD:

PPSV23

Age	Years Since Vaccination					
	0	1	2	3	4	5
...						
70	75.7					
71	74.8					
72	73.9					
73	73.1					
74	72.2					
75	71.3					
...						

PCV13

Age	Years Since Vaccination					
	0	1	2	3	4	5
...						
70	45.8					
71	45.5					
72	45.2					
73	45.0	45.0	45.0	45.0	45.0	
74	44.7					
75	44.4					
...						

For each age, VE-PCV13 was assumed to be durable:

PPSV23

Age	Years Since Vaccination					
	0	1	2	3	4	5
...						
70	75.7					
71	74.8					
72	73.9					
73	73.1					
74	72.2					
75	71.3					
...						

PCV13

Age	Years Since Vaccination					
	0	1	2	3	4	5
...						
70	45.8	45.8	45.8	45.8	45.8	
71	45.5	45.5	45.5	45.5	45.5	
72	45.2	45.2	45.2	45.2	45.2	
73	45.0	45.0	45.0	45.0	45.0	
74	44.7	44.7	44.7	44.7	44.7	
75	44.4	44.4	44.4	44.4	44.4	
...						

To extrapolate age-specific values beyond 5th-year of modeling horizon, 50% of observed decrease in VE-PPSV23 between respective 1-year time increments was applied to PCV13 values (staggered by 5-year assumed durability):

PPSV23

	Years Since Vaccination					
Age	0	1	2	3	4	5
...						
70	75.7	71.1				
71	74.8	70.0				
72	73.9	68.9				
73	73.1	67.8				
74	72.2	66.7				
75	71.3	65. 6				
...						

PCV13

	Years Since Vaccination					
Age	0	1	2	3	4	5
...						
70	45.8	45.8	45.8	45.8	45.8	44.4
71	45.5	45.5	45.5	45.5	45.5	44.0
72	45.2	45.2	45.2	45.2	45.2	43.7
73	45.0	45.0	45.0	45.0	45.0	43.4
74	44.7	44.7	44.7	44.7	44.7	43.0
75	44.4	44.4	44.4	44.4	44.4	42.6
...						

VE-PCV13 is assumed to be 0 by—at latest—year 16 of modeling horizon. For high-risk persons, VE-PCV13 was assumed to be 65% of corresponding values for low/moderate-risk persons based on Klugman et al. (2003) [5] (13% vs 20% VE):

Table 4. Efficacy of the Vaccine against First Episodes of Radiologically Confirmed Pneumonia.*

Variable	Vaccinated Group	Control Group	P Value	Vaccine Efficacy (95% CI)
	no. of episodes			%
HIV-negative children	169	212	0.03	20 (2 to 35)
HIV-positive children	182	209	0.19	13 (−7 to 29)
All children	356	428	0.01	17 (4 to 28)

* CI denotes confidence interval, and HIV human immunodeficiency virus.

Finally, to convert the effectiveness estimates from pneumococcal CAP to all-cause CAP, each value was multiplied by 0.08 (assuming the 8% of all-cause CAP was attributable to VT-NBP).

REFERENCES

1. Smith K. J., Zimmerman R. K., Lin C. J., Nowalk M. P., Ko F. S., McEllistrem M. C., Roberts M. S. Alternative strategies for adult pneumococcal polysaccharide vaccination: a cost-effectiveness analysis. Vaccine. 2008 Mar 10; 26(11): 1420-31.

2. Shapiro E. D., Berg A. T., Austrian R., Schroeder D., Parcells V., Margolis A., Adair R. K., Clemens J. D. The protective efficacy of polyvalent pneumococcal polysaccharide vaccine. N Engl J Med. 1991 Nov 21; 325(21): 1453-60.

3. Fry A. M., Zell E. R., Schuchat A., Butler J. C., Whitney C. G. Comparing potential benefits of new pneumococcal vaccines with the current polysaccharide vaccine in the elderly. Vaccine. 2002 Dec 13; 21(3-4): 303-11.

4. Bonten M. J., Huijts S. M., Bolkenbaas M., Webber C., Patterson S., Gault S., van Werkhoven C. H., van Deursen A. M., Sanders E. A., et al. Polysaccharide conjugate vaccine against pneumococcal pneumonia in adults. N Engl J Med. 2015 Mar 19; 372(12): 1114-25.

5. Klugman K. P., Madhi S. A., Huebner R. E., Kohberger R., Mbelle N., Pierce N., Vaccine Trialists Group. A trial of a 9-valent pneumococcal conjugate vaccine in children with and those without HIV infection. N Engl J Med. 2003 Oct 02; 349(14): 1341-8.

6. Cho B. H., Stoecker C., Link-Gelles R., Moore M. R. Cost-effectiveness of administering 13-valent pneumococcal conjugate vaccine in addition to 23-valent pneumococcal polysaccharide vaccine to adults with immunocompromising conditions. Vaccine. 2013 Dec 05; 31(50): 6011-21.

7. Jackson L. A., Neuzil K. M., Yu O., Benson P., Barlow W. E., Adams A. L., Hanson C. A., Mahoney L. D., Shay D. K., et al. Effectiveness of pneumococcal polysaccharide vaccine in older adults. N Engl J Med. 2003 May 01; 348(18): 1747-55.

8. Smith K. J., Wateska A. R., Nowalk M. P., Raymund M., Lee B. Y., Zimmerman R. K. Modeling of cost effectiveness of pneumococcal conjugate vaccination strategies in U.S. older adults. Am J Prev Med. 2013 Apr; 44(4): 373-81.
