# Supplementary material for: Estimating the cost-effectiveness of a sequential pneumococcal vaccination program for adults in Germany
Source: PLoS One. 2018 May 24;13(5):e0197905. doi: 10.1371/journal.pone.0197905 (PMC5967715; doi:10.1371/journal.pone.0197905)
Supplement: S1 Table — (PDF) [file pone.0197905.s002.pdf]

S1 Table. Model fits for serotype distribution IPD over time (incl. prognosis for next 5 years)

Model[a]	Formula	AIC	BIC	Pred_2017	Pred_2018	Pred_2019	Pred_2020	Pred_2021
**PCV13-7: 16-49**								
1	*a * exp(b * Year)*	*-21.17*	*-21.79*	*0.218*	*0.183*	*0.153*	*0.128*	*0.107*
2	exp(a + b / Year)	-19.71	-20.34	0.235	0.211	0.192	0.176	0.163
3	a * Year^b	-20.71	-21.34	0.227	0.197	0.173	0.153	0.136
4	a * Year^b * exp(c * Year)	-19.2	-20.03	0.215	0.178	0.147	0.121	0.099
5	a * b * log(Year)	na	na	na	na	na	na	na
6	d + a * Year^b * exp(c * Year)	na	na	na	na	na	na	na
7	c + a * exp(b * Year)	na	na	na	na	na	na	na
**PCV13-7: 50-60**								
1	a * exp(b * Year)	-19.36	-19.99	0.203	0.169	0.141	0.118	0.098
2	*exp(a + b / Year)*	*-21.73*	*-22.36*	*0.216*	*0.193*	*0.175*	*0.16*	*0.147*
3	a * Year^b	-20.63	-21.26	0.209	0.181	0.158	0.139	0.123
4	a * Year^b * exp(c * Year)	na	na	na	na	na	na	na
5	a * b * log(Year)	na	na	na	na	na	na	na
6	d + a * Year^b * exp(c * Year)	na	na	na	na	na	na	na
**PCV13-7: 60-75**								
1	a * exp(b * Year)	-28.08	-28.71	0.268	0.239	0.213	0.19	0.17
2	exp(a + b / Year)	-28.78	-29.4	0.279	0.26	0.244	0.23	0.219
3	*a * Year^b*	*-29*	*-29.62*	*0.273*	*0.25*	*0.229*	*0.211*	*0.196*
4	a * Year^b * exp(c * Year)	-27.11	-27.94	0.276	0.255	0.237	0.222	0.209
5	a * b * log(Year)	na	na	na	na	na	na	na

6	d + a * Year^b * exp(c * Year)	na	na	na	na	na	na	na
7	c + a * exp(b * Year)	-27.43	-28.26	0.277	0.257	0.241	0.229	0.219
**PCV13-7: 75+**								
1	*a * exp(b * Year)*	*-36.2*	*-36.82*	*0.263*	*0.24*	*0.219*	*0.2*	*0.183*
2	exp(a + b / Year)	-31.22	-31.84	0.271	0.256	0.244	0.233	0.224
3	a * Year^b	-33.77	-34.4	0.267	0.249	0.232	0.218	0.205
4	a * Year^b * exp(c * Year)	-35.61	-36.45	0.258	0.231	0.206	0.182	0.161
5	a * b * log(Year)	na	na	na	na	na	na	na
6	d + a * Year^b * exp(c * Year)	na	na	na	na	na	na	na
7	c + a * exp(b * Year)	na	na	na	na	na	na	na

[a] To predict indirect effects of PCV13 vaccination for the next 5 years, we analyzed data from the National Reference Laboratory (NRZ) on Streptococcal Diseases (38). Data show the observed ST-distribution for the periods 2010/11-2015/16 for PCV7 und PCV13. We tested different models to evaluate the effect of PCV13 and used the model with the best fit in the age group. Model fits confirmed our assumption that the final level of indirect effect was reached after 5 years.
