# Supplementary material for: Estimating the cost-effectiveness of a sequential pneumococcal vaccination program for adults in Germany
Source: PLoS One. 2018 May 24;13(5):e0197905. doi: 10.1371/journal.pone.0197905 (PMC5967715; doi:10.1371/journal.pone.0197905)
Supplement: S2 Table — (PDF) [file pone.0197905.s003.pdf]

S2 Table. Development of herd effect by year of modelling (cumulative in %) based on ST-specific IPD data

Year of modelling	Age group (years)a					Source
	18-49	50-59	60-64	65-74	75-99	
1	0	0	0	0	0	[1] and S1 Table
2	81.3	88.2	80.4	80.4	74.8	
3	88.7	92.8	88.0	88.0	84.1	
4	94.8	96.7	94.6	94.6	92.5	
5+	100	100	100	100	100	

[a] Effects are set equal for all risk groups
