# Supplementary material for: Estimating the cost-effectiveness of a sequential pneumococcal vaccination program for adults in Germany
Source: PLoS One. 2018 May 24;13(5):e0197905. doi: 10.1371/journal.pone.0197905 (PMC5967715; doi:10.1371/journal.pone.0197905)
Supplement: S3 Table — (PDF) [file pone.0197905.s004.pdf]

S3 Table. Distribution of PSA parameters

Parameters	Distribution					Source
**Incidence**	**Cases per 100,000 in 18-49y.**	**Beta Distribution[a]**				
		**No. of Cases in Cohort (α)**		**No. of Cases in Cohort (β)**		
Bacteremia	2	2,489	291	26,997,511	15,039,709	[1]
Meningitis	0.1	131	21	26,999,869	15,039,979	
Inpatient Pneumonia	198	89,785	133,440	25,802,042	67,334,756	
Outpatient Pneumonia	280	4,536	189,034	1,435,464	67,279,162	
**Effectiveness of PCV13**	**Vaccine-Type Effectiveness[b]**	**Beta Distribution[a]**				
		**No. of Cases in Cohort (α)**		**No. of Cases in Cohort (β)**		
Bacteremia/Meningitis						
Vaccine	76%	7	-	42,233	-	[2]
Control	-	28	-	42,228	-	
Inpatient Pneumonia						
Vaccine	45%	33	-	42,207	-	
Control	-	60	-	42,196	-	
Outpatient Pneumonia						
Vaccine	45%	33	-	42,207	-	
Control	-	60	-	42,496	-	
**Reduction in Effectiveness in High-Risk vs Low/Mod-Risk**	**Reduction**	**No. of Cases in Cohort (α)**		**No. of Cases in Cohort (β)**		
Vaccine	65%	9	-	18,927	-	[3]
Control	-	26	-	18,941	-	
**Effectiveness of PPSV23**	**Effectiveness in 18-49y.**	**Beta Distribution[a]**				

		No. of Cases in Cohort (α)		No. of Cases in Cohort (β)		
Bacteremia/Meningitis						
Low-risk						
Vaccine	93%	7	-	118	-	[4-6]
Control	-	100	-	25	-	
High-risk						
Post-vaccination	21%	15	-	160	-	
Pre-vaccination	-	19	-	156	-	
**Mortality**	**Deaths per 100 in 18-49y.**	**Beta Distribution[a]**				
		**No. of Cases in Cohort (α)**		**No. of Cases in Cohort (β)**		
Bacteremia	20	49	4,542	268	17,756	[7]
Meningitis	20	49	4,542	268	17,756	
Inpatient Pneumonia	14	710	54,758	5,508	333,600	[8]
**Medical Costs**	**Costs per Cases in 18-49y.**	**Log-Normal Distribution[a]**				
		**Mean**		**Standard Error**		
Bacteremia	€7,914	€27,376	€7,914	€480	€593	[9]
Meningitis	€4,648	€27,376	€4,648	€480	€102	
Inpatient Pneumonia	€3,415	€11,638	€3,415	€124	€173	
Outpatient Pneumonia	€71	€97	€71	€7	€0	[10-13]

[a] Distribution of values characterizing uncertainty around point estimate for youngest age group.

[b] Effectiveness estimates are based on data from CAPiTA trial and correspond to the mean age of subjects, 73 years.

2
