# Supplementary material for: Estimating the cost-effectiveness of a sequential pneumococcal vaccination program for adults in Germany
Source: PLoS One. 2018 May 24;13(5):e0197905. doi: 10.1371/journal.pone.0197905 (PMC5967715; doi:10.1371/journal.pone.0197905)
Supplement: S4 Table — (PDF) [file pone.0197905.s005.pdf]

S4 Table. Expected lifetime disease-related cases, deaths, and costs in German adults #1-#4

**Scenario** (description)	**#1** (sequential for all risk groups)				**#2** (LR according to STIKO, sequential only for MR and HR)				**#3** (LR and MR initial vaccination with PCV13, HR sequential)				**#4** (#1 with immediate waning for PCV13)			
	**Current**	**Hypothetical**	**Δ**	**95% CI**	**Current**	**Hypothetical**	**Δ**	**95% CI**	**Current**	**Hypothetical**	**Δ**	**95% CI**	**Current**	**Hypothetical**	**Δ**	**95% CI**
**Population-Level Results**																
No. of Cases																
IPD (in thousands)	85.008	84.870	-0.138	(-1.866, 1.392)	85.283	85.173	-0.110	(-1.854, 1.432)	85.008	84.936	-0.072	(-13.284, 12.708)	85.425	85.336	-0.089	(-1.904, 1.460)
NBP (in millions)																
Requiring Inpatient Care	16.546	16.501	-0.045	(-0.057, -0.017)	16.547	16.504	-0.043	(-0.056, -0.017)	16.546	16.501	-0.044	(-0.134, 0.047)	16.552	16.527	-0.024	(-0.038, 0.001)
Requiring Outpatient Care	23.278	23.245	-0.033	(-0.062, -0.003)	23.279	23.249	-0.030	(-0.058, 0.001)	23.278	23.245	-0.033	(-0.142, 0.079)	23.280	23.265	-0.015	(-0.039, 0.011)
No. of Deaths (in millions)	2.857	2.850	-0.007	(-0.015, 0.001)	2.859	2.852	-0.007	(-0.014, 0.001)	2.857	2.851	-0.006	(-0.033, 0.021)	2.860	2.856	-0.004	(-0.011, 0.004)
Total Costs (in billions)																
Medical Care	30.671	30.534	-0.137	(-0.177, -0.090)	30.594	30.463	-0.131	(-0.171, -0.086)	30.671	30.554	-0.117	(-0.303, 0.018)	65.914	65.793	-0.122	(-0.172, -0.068)
Non-Medical Care	3.835	3.810	-0.025	(-0.077, 0.026)	3.842	3.816	-0.026	(-0.077, 0.027)	3.835	3.817	-0.018	(-0.089, 0.050)	3.881	3.816	-0.065	(-0.189, 0.025)

Vaccination	0.505	1.082	0.577	(0.576, 0.579)	0.505	0.967	0.462	(0.461, 0.464)	0.505	0.789	0.284	(0.283, 0.285)	0.505	1.082	0.577	(0.576, 0.579)
Total																
Medical + Vaccination	31.176	31.616	0.440	(0.399, 0.487)	31.099	31.430	0.331	(0.291, 0.376)	31.176	31.343	0.167	(-0.018, 0.302)	66.419	66.875	0.456	(0.406, 0.510)
Medical + Non-Medical + Vaccination	35.011	35.426	0.415	(0.335, 0.494)	34.941	35.246	0.305	(0.223, 0.387)	35.011	35.160	0.149	(-0.042, 0.281)	70.300	70.690	0.391	(0.252, 0.508)
**Patient-Level Results**																
Total Costs																
Medical Care	454.61	452.58	-2.03	(-2.630, -1.336)	453.46	451.52	-1.95	(-2.532, -1.274)	454.61	452.87	-1.74	(-4.486, 0.267)	976.97	975.17	-1.80	(-7.040, -2.440)
Non-Medical Care	56.85	56.47	-0.38	(-1.139, 0.382)	56.95	56.56	-0.39	(-1.142, 0.396)	56.85	56.57	-0.27	(-1.324, 0.746)	57.52	56.56	-0.96	(-4.411, 0.482)
Vaccination	7.48	16.04	8.56	(8.541, 8.580)	7.48	14.33	6.85	(6.833, 6.870)	7.48	11.69	4.21	(4.197, 4.228)	7.48	16.04	8.56	(8.540, 8.576)
Total																
Medical + Vaccination	462.09	468.61	6.53	(5.921, 7.224)	460.94	465.85	4.90	(4.307, 5.573)	462.09	464.56	2.48	(-0.270, 4.477)	984.45	991.20	6.76	(6.018, 7.556)
Medical + Non-Medical + Vaccination	518.93	525.08	6.15	(4.963, 7.321)	517.89	522.41	4.52	(3.305, 5.733)	518.93	521.13	2.20	(-0.622, 4.159)	1,041.97	1,047.76	5.79	(3.730, 7.523)

Life-Years (discounted)	18.7107	18.7114	0.0007	(-0.0013, 0.0030)	18.7107	18.7114	0.0007	(-0.0013, 0.0032)	18.7107	18.7113	0.0007	(-0.0030, 0.0043)	18.7105	18.7109	0.0004	(-0.0024, 0.0026)
QALY (discounted)	15.1794	15.1798	0.0004	(-0.0009, 0.0021)	15.1794	15.1799	0.0004	(-0.0009, 0.0022)	15.1794	15.1798	0.0004	(-0.0020, 0.0029)	15.1793	15.1795	0.0002	(-0.0014, 0.0015)
**Healthcare System Perspective**																
Cost per Life-Year Gained	€ 8,964				€ 7,013				€ 3,662				€ 16,879			
Cost per QALY Gained	€ 14,881				€ 11,584				€ 5,828				€ 29,635			
Societal Perspective																
Cost per Life-Year Gained	€ 8,447				€ 6,459				€ 3,258				€ 14,470			
Cost per QALY Gained	€ 14,023				€ 10,667				€ 5,186				€ 25,406			

QALY: quality-adjusted life year

Note: Low-risk is specified as immunocompetent patients without any chronic medical conditions, moderate-risk describes immunocompetent patients with at least one chronic medical condition and high-risk represent immunocompromised/immunosuppressed patients, with or without chronic medical conditions (congenital or acquired).

Healthcare system perspective includes medical and vaccination costs; societal perspective includes medical, non-medical, and vaccination costs.
