# Supplementary material for: Estimating the cost-effectiveness of a sequential pneumococcal vaccination program for adults in Germany
Source: PLoS One. 2018 May 24;13(5):e0197905. doi: 10.1371/journal.pone.0197905 (PMC5967715; doi:10.1371/journal.pone.0197905)
Supplement: S5 Table — (PDF) [file pone.0197905.s006.pdf]

S5 Table. Expected lifetime disease-related cases, deaths, and costs in German adults #5-#7

**Scenario** (description)	**#5** (#1 with 64% NBP Effectiveness PPSV23)				**#6** (#1 with 50% revaccination rate)				**#7** (#2 with 50% revaccination rate)			
	**Current**	**Hypothetical**	**Δ**	**95% CI**	**Current**	**Hypothetical**	**Δ**	**95% CI**	**Current**	**Hypothetical**	**Δ**	**95% CI**
**Population-Level Results**												
No, of Cases												
IPD (in thousands)	85.369	85.321	-0.048	(-1.505, 1.075)	85.423	85.298	-0.125	(-1.929, 1.487)	84.910	84.768	-0.142	(-1.882, 1.465)
NBP (in millions)												
Requiring Inpatient Care	16.540	16.509	-0.031	(-0.043, -0.018)	16.547	16.503	-0.044	(-0.057, -0.016)	16.546	16.503	-0.043	(-0.056, -0.013)
Requiring Outpatient Care	23.257	23.211	-0.045	(-0.067, -0.026)	23.278	23.245	-0.033	(-0.064, -0.003)	23.276	23.247	-0.030	(-0.059, -0.001)
No, of Deaths (in millions)	2.859	2.855	-0.004	(-0.010, 0.002)	2.858	2.851	-0.008	(-0.015, 0.000)	2.858	2.851	-0.007	(-0.014, 0.001)
Total Costs (in billions)												
Medical Care	30.634	30.538	-0.096	(-0.201, 0.014)	30.601	30.465	-0.136	(-0.176, -0.085)	30.638	30.507	-0.132	(-0.173, -0.078)
Non-Medical Care	3.831	3.812	-0.019	(-0.058, 0.018)	3.841	3.815	-0.026	(-0.076, 0.028)	3.842	3.815	-0.027	(-0.078, 0.029)
Vaccination	0.505	1.082	0.577	(0.576, 0.578)	0.505	1.082	0.577	(0.576, 0.579)	0.505	0.967	0.462	(0.461, 0.463)

Total												
Medical + Vaccination	31.139	31.620	0.481	(0.376, 0.591)	31.106	31.547	0.442	(0.401, 0.492)	31.143	31.473	0.330	(0.290, 0.383)
Medical + Non-Medical + Vaccination	34.969	35.431	0.462	(0.357, 0.571)	34.947	35.362	0.415	(0.336, 0.494)	34.985	35.288	0.303	(0.224, 0.385)
**Patient-Level Results**												
Total Costs												
Medical Care	454.05	452.63	-1.42	(-7.638, -0.114)	453.56	451.55	-2.01	(-2.609, -1.263)	454.11	452.16	-1.95	(-2.557, -1.160)
Non-Medical Care	56.78	56.49	-0.28	(-1.398, 0.337)	56.93	56.54	-0.39	(-1.121, 0.408)	56.95	56.54	-0.40	(-1.159, 0.430)
Vaccination	7.48	16.04	8.56	(8.544, 8.568)	7.48	16.04	8.56	(8.542, 8.576)	7.48	14.33	6.85	(6.835, 6.866)
Total												
Medical + Vaccination	461.53	468.66	7.13	(5.573, 8.763)	461.04	467.59	6.55	(5.939, 7.291)	461.59	466.49	4.90	(4.293, 5.680)
Medical + Non-Medical + Vaccination	518.31	525.16	6.85	(5.284, 8.466)	517.98	524.13	6.16	(4.977, 7.315)	518.54	523.03	4.49	(3.317, 5.713)
Life-Years (discounted)	18.7111	18.7114	0.0003	(-0.0014, 0.0020)	18.7106	18.7113	0.0007	(-0.0014, 0.0031)	18.7108	18.7114	0.0006	(-0.0014, 0.0030)
QALY (discounted)	16.2024	16.2026	0.0002	(-0.0021, 0.0024)	15.1794	15.1798	0.0004	(-0.0010, 0.0021)	15.1795	15.1798	0.0004	(-0.0010, 0.0021)

**Healthcare System Perspective**			
Cost per Life-Year Gained	€ 23,061	€ 9,527	€ 7,946
Cost per QALY Gained	€ 29,617	€ 5,828	€ 13,699
**Societal Perspective**			
Cost per Life-Year Gained	€ 22,146	€ 8,960	€ 7,290
Cost per QALY Gained	€ 28,441	€ 14,898	€ 12,570

QALY: quality-adjusted life year

Note: Low-risk is specified as immunocompetent patients without any chronic medical conditions, moderate-risk describes immunocompetent patients with at least one chronic medical condition and high-risk represent immunocompromised/immunosuppressed patients, with or without chronic medical conditions (congenital or acquired). Healthcare system perspective includes medical and vaccination costs; societal perspective includes medical, non-medical, and vaccination costs.
