# Supplementary material for: Estimating the cost-effectiveness of a sequential pneumococcal vaccination program for adults in Germany
Source: PLoS One. 2018 May 24;13(5):e0197905. doi: 10.1371/journal.pone.0197905 (PMC5967715; doi:10.1371/journal.pone.0197905)
Supplement: S6 Table — (PDF) [file pone.0197905.s007.pdf]

S6 Table. Expected lifetime disease-related cases, deaths, and costs in German adults <60 years (#1-#4)

**Scenario** (description)	**#1** (sequential for all risk groups)				**#2** (LR according to STIKO, sequential only for MR and HR)				**#3** (LR and MR initial vaccination with PCV13, HR sequential)				**#4** (#1 with immediate waning for PCV13)			
	**Cur-rent**	**Hypothet-ical**	**Δ**	**95% CI**	**Current**	**Hypothet-ical**	**Δ**	**95% CI**	**Current**	**Hypothet-ical**	**Δ**	**95% CI**	**Current**	**Hypothet-ical**	**Δ**	**95% CI**
**Population-Level Results**																
No. of Cases																
IPD (in thou-sands)	60.462	60.370	-0.093	(-1.153, 1.069)	60.667	60.578	-0.089	(-1.183, 1.054)	60.462	60.523	0.060	(-9.160, 8.868)	60.736	60.648	-0.088	(-1.316, 1.257)
NBP (in millions)																
Requiring In-patient Care	10.069	10.063	-0.006	(-0.015, 0.005)	10.069	10.064	-0.006	(-0.016, 0.005)	10.069	10.063	-0.005	(-0.066, 0.051)	10.068	10.064	-0.005	(-0.015, 0.006)
Requiring Out-patient Care	14.174	14.163	-0.011	(-0.024, 0.004)	14.175	14.164	-0.011	(-0.025, 0.006)	14.174	14.164	-0.011	(-0.076, 0.055)	14.175	14.169	-0.005	(-0.019, 0.010)
No. of Deaths (in millions)	1.541	1.540	0.000	(-0.003, 0.003)	1.541	1.541	-0.001	(-0.003, 0.002)	1.541	1.541	0.000	(-0.015, 0.015)	1.541	1.540	-0.001	(-0.003, 0.003)
Total Costs (in billions)																
Medical Care	14.287	14.267	-0.020	(-0.036, -0.004)	14.251	14.231	-0.020	(-0.036, -0.003)	14.287	14.276	-0.011	(-2.013, 1.816)	30.735	30.704	-0.030	(-0.049, -0.012)
Non-Medical Care	3.156	3.136	-0.020	(-0.069, 0.030)	3.163	3.142	-0.021	(-0.070, 0.030)	3.156	3.143	-0.013	(-0.076, 0.054)	3.200	3.139	-0.061	(-0.185, 0.026)
Vaccination	0.193	0.441	0.247	(0.247,	0.193	0.441	0.247	(0.247,	0.193	0.315	0.122	(0.121,	0.193	0.441	0.247	(0.247,

				0.248)				0.248)				0.122)				0.248)
Total																
Medical + Vaccination	14.480	14.708	0.228	(0.212, 0.244)	14.444	14.672	0.228	(0.211, 0.245)	14.480	14.591	0.111	(-1.891, 1.938)	30.928	31.145	0.217	(0.198, 0.235)
Medical + Non-Medical + Vaccination	17.636	17.844	0.208	(0.154, 0.263)	17.607	17.814	0.207	(0.152, 0.263)	17.636	17.734	0.098	(-1.906, 1.935)	34.128	34.284	0.156	(0.025, 0.247)
**Patient-Level Results**																
Total Costs																
Medical Care	311.89	311.46	-0.43	(-0.79, -0.08)	311.10	310.67	-0.43	(-0.79, -0.07)	311.89	311.65	-0.24	(-43.94, 39.65)	670.96	670.29	-0.67	(-1.08, -0.27)
Non-Medical Care	68.90	68.46	-0.44	(-1.52, 0.66)	69.05	68.59	-0.46	(-1.53, 0.66)	68.90	68.62	-0.28	(-1.66, 1.17)	69.85	68.53	-1.33	(-4.04, 0.57)
Vaccination	4.22	9.62	5.40	(5.39, 5.42)	4.22	9.62	5.40	(5.39, 5.42)	4.22	6.88	2.66	(2.65, 2.67)	4.22	9.62	5.40	(5.38, 5.42)
Total																
Medical + Vaccination	316.10	321.08	4.97	(4.62, 5.33)	315.32	320.29	4.97	(4.62, 5.34)	316.10	318.52	2.42	(-41.27, 42.31)	675.17	679.91	4.74	(4.32, 5.12)
Medical + Non-Medical + Vaccination	385.01	389.54	4.53	(3.37, 5.75)	384.37	388.88	4.51	(3.32, 5.73)	385.01	387.14	2.13	(-41.61, 42.24)	745.03	748.44	3.41	(0.54, 5.39)
Life-Years (dis-counted)	22.6801	22.6802	0.0001	(-0.0020, 0.0022)	22.6801	22.6803	0.0002	(-0.0021, 0.0025)	22.6801	22.6802	0.0001	(-0.0039, 0.0041)	22.6800	22.6800	0.0000	(-0.0020, 0.0020)
QALY (discount-	19.044	19.0441	0.0001	(-0.0013,	19.0440	19.0442	0.0001	(-0.0013,	19.0440	19.0441	0.0001	(-0.0025,	19.0440	19.0440	0.0000	(-0.0012,

ed)	0			0.0015)				0.0017)				0.0028)				0.0014)
**Healthcare System Perspective**																
Cost per Life-Year Gained	€ 34,154				€ 26,510				€ 31,506				€ 128,026			
Cost per QALY Gained	€ 50,497				€ 37,328				€ 30,578				€ 4,478,002			
Societal Perspective																
Cost per Life-Year Gained	€ 31,139				€ 24,040				€ 27,811				€ 92,195			
Cost per QALY Gained	€ 46,039				€ 33,850				€ 26,992				€ 3,224,738			

QALY: quality-adjusted life year

Note: Low-risk is specified as immunocompetent patients without any chronic medical conditions, moderate-risk describes immunocompetent patients with at least one chronic medical condition and high-risk represent immunocompromised/immunosuppressed patients, with or without chronic medical conditions (congenital or acquired).

Healthcare system perspective includes medical and vaccination costs; societal perspective includes medical, non-medical, and vaccination costs.

2
