# Supplementary material for: Estimating the cost-effectiveness of a sequential pneumococcal vaccination program for adults in Germany
Source: PLoS One. 2018 May 24;13(5):e0197905. doi: 10.1371/journal.pone.0197905 (PMC5967715; doi:10.1371/journal.pone.0197905)
Supplement: S7 Table — (PDF) [file pone.0197905.s008.pdf]

S7 Table. Expected lifetime disease-related cases, deaths, and costs in German adults <60 years (#5-#8)

**Scenario** (description)	**#5** (#1 with 64% NBP Effectiveness PPSV23)				**#6** (#1 with 50% revaccination rate)				**#7** (#2 with 50% revaccination rate)			
	**Cur-rent**	**Hypothet-ical**	**Δ**	**95% CI**	**Current**	**Hypothet-ical**	**Δ**	**95% CI**	**Current**	**Hypothet-ical**	**Δ**	**95% CI**
No. of Cases												
IPD (in thou-sands)	60.870	60.814	-0.056	(-0.837, 0.848)	60.777	60.674	-0.102	(-1.213, 1.090)	60.400	60.300	-0.100	(-1.150, 1.140)
NBP (in millions)												
Requiring In-patient Care	10.068	10.063	-0.005	(-0.013, 0.003)	10.070	10.064	-0.005	(-0.015, 0.007)	10.068	10.063	-0.006	(-0.015, 0.006)
Requiring Outpatient Care	14.174	14.167	-0.007	(-0.017, 0.003)	14.174	14.164	-0.010	(-0.025, 0.005)	14.173	14.163	-0.011	(-0.025, 0.004)
No. of Deaths (in millions)	1.542	1.541	-0.001	(-0.003, 0.001)	1.541	1.540	-0.001	(-0.004, 0.002)	1.541	1.540	-0.001	(-0.004, 0.002)
Total Costs (in billions)												
Medical Care	14.278	14.260	-0.018	(-0.091, 0.060)	14.255	14.235	-0.019	(-0.035, -0.002)	14.271	14.251	-0.020	(-0.036, -0.003)
Non-Medical Care	3.154	3.139	-0.016	(-0.052, 0.021)	3.162	3.141	-0.021	(-0.068, 0.032)	3.163	3.141	-0.022	(-0.071, 0.033)
Vaccination	0.193	0.441	0.247	(0.247, 0.248)	0.193	0.441	0.247	(0.247, 0.248)	0.193	0.441	0.247	(0.247, 0.248)
Total												

Medical + Vaccination	14.472	14.701	0.229	(0.156, 0.308)	14.448	14.676	0.228	(0.212, 0.245)	14.464	14.691	0.228	(0.211, 0.245)
Medical + Non-Medical + Vaccination	17.626	17.840	0.214	(0.141, 0.291)	17.610	17.817	0.207	(0.156, 0.263)	17.627	17.832	0.205	(0.153, 0.266)
**Patient-Level Results**												
Total Costs												
Medical Care	311.71	311.31	-0.40	(-1.99, 1.32)	311.19	310.77	-0.42	(-0.77, -0.05)	311.53	311.10	-0.43	(-0.79, -0.06)
Non-Medical Care	68.86	68.52	-0.34	(-1.13, 0.45)	69.03	68.57	-0.46	(-1.48, 0.69)	69.05	68.57	-0.48	(-1.54, 0.72)
Vaccination	4.22	9.62	5.40	(5.39, 5.41)	4.22	9.62	5.40	(5.39, 5.42)	4.22	9.62	5.40	(5.39, 5.42)
Total												
Medical + Vaccination	315.92	320.93	5.01	(3.41, 6.72)	315.40	320.38	4.98	(4.64, 5.35)	315.75	320.72	4.97	(4.61, 5.35)
Medical + Non-Medical + Vaccination	384.78	389.45	4.66	(3.08, 6.36)	384.43	388.96	4.53	(3.41, 5.74)	384.80	389.29	4.49	(3.34, 5.81)
Life-Years (discounted)	22.6802	22.6803	0.0001	(-0.0013, 0.0016)	22.6800	22.6802	0.0001	(-0.0020, 0.0023)	22.6801	22.6802	0.0001	(-0.0021, 0.0023)
QALY (discounted)	19.7573	19.7574	0.0001	(-0.0010, 0.0014)	19.0440	19.0441	0.0001	(-0.0012, 0.0016)	19.0441	19.0441	0.0001	(-0.0013, 0.0017)
**Healthcare System Perspective**												

Cost per Life-Year Gained	€ 43,230	€ 33,262	€ 35,527
Cost per QALY Gained	€ 55,366	€ 50,526	€ 54,658
**Societal Perspective**			
Cost per Life-Year Gained	€ 40,268	€ 30,207	€ 32,062
Cost per QALY Gained	€ 51,572	€ 45,884	€ 49,328

QALY: quality-adjusted life year

Note: Low-risk is specified as immunocompetent patients without any chronic medical conditions, moderate-risk describes immunocompetent patients with at least one chronic medical condition and high-risk represent immunocompromised/immunosuppressed patients, with or without chronic medical conditions (congenital or acquired). Healthcare system perspective includes medical and vaccination costs; societal perspective includes medical, non-medical, and vaccination costs.
