# Supplementary material for: Estimating the cost-effectiveness of a sequential pneumococcal vaccination program for adults in Germany
Source: PLoS One. 2018 May 24;13(5):e0197905. doi: 10.1371/journal.pone.0197905 (PMC5967715; doi:10.1371/journal.pone.0197905)
Supplement: S8 Table — (PDF) [file pone.0197905.s009.pdf]

S8 Table. Expected lifetime disease-related cases, deaths, and costs in German adults ≥60 years (#1-#4)

**Scenario** (description)	**#1** (sequential for all risk groups)				**#2** (LR according to STIKO, sequential only for MR and HR)				**#3** (LR and MR initial vaccination with PCV13, HR sequential)				**#4** (#1 with immediate waning for PCV13)			
	**Current**	**Hypothetical**	**Δ**	**95% CI**	**Current**	**Hypothetical**	**Δ**	**95% CI**	**Current**	**Hypothetical**	**Δ**	**95% CI**	**Current**	**Hypothetical**	**Δ**	**95% CI**
**Population-Level Results**																
No. of Cases																
IPD (in thousands)	24.546	24.500	-0.045	(-1.132, 1.142)	24.616	24.594	-0.022	(-1.087, 1.111)	24.546	24.413	-0.133	(-4.098, 3.816)	24.689	24.688	0.000	(-1.062, 1.082)
NBP (in millions)																
Requiring Inpatient Care	6.477	6.438	-0.039	(-0.051, -0.020)	6.478	6.440	-0.037	(-0.050, -0.020)	6.477	6.438	-0.039	(-0.075, -0.004)	6.483	6.464	-0.020	(-0.032, -0.003)
Requiring Outpatient Care	9.104	9.082	-0.022	(-0.046, -0.005)	9.104	9.085	-0.019	(-0.041, -0.001)	9.104	9.081	-0.023	(-0.069, 0.020)	9.106	9.096	-0.010	(-0.027, 0.006)
No. of Deaths (in millions)	1.317	1.310	-0.007	(-0.014, 0.000)	1.317	1.311	-0.006	(-0.013, 0.000)	1.317	1.310	-0.006	(-0.019, 0.007)	1.319	1.316	-0.003	(-0.010, 0.003)
Total Costs (in billions)																
Medical Care	16.385	16.267	-0.117	(-0.154, -0.075)	16.344	16.232	-0.112	(-0.148, -0.072)	16.385	16.279	-0.106	(-2.418, 1.988)	35.180	35.088	-0.091	(-0.139, -0.040)
Non-Medical Care	0.679	0.674	-0.005	(-0.009, -0.001)	0.679	0.674	-0.005	(-0.009, -0.001)	0.679	0.674	-0.005	(-0.013, 0.003)	0.681	0.677	-0.004	(-0.019, 0.006)
Vaccination	0.312	0.641	0.330	(0.329,	0.312	0.526	0.215	(0.214,	0.312	0.474	0.162	(0.162,	0.312	0.641	0.330	(0.329,

				0.331)				0.216)				0.163)				0.331)
Total																
Medical + Vaccination	16.696	16.909	0.213	(0.175, 0.255)	16.655	16.758	0.103	(0.067, 0.143)	16.696	16.753	0.056	(-2.256, 2.150)	35.491	35.730	0.239	(0.191, 0.290)
Medical + Non-Medical + Vaccination	17.375	17.583	0.207	(0.170, 0.251)	17.334	17.432	0.098	(0.061, 0.139)	17.375	17.426	0.051	(-2.269, 2.148)	36.172	36.406	0.234	(0.187, 0.287)
**Patient-Level Results**																
Total Costs																
Medical Care	756.42	751.00	-5.42	(-7.11, -3.45)	754.52	749.37	-5.15	(-6.82, -3.30)	756.42	751.52	-4.90	(-111.63, 91.76)	1,624.10	1,619.89	-4.21	(-6.41, -1.85)
Non-Medical Care	31.35	31.10	-0.24	(-0.42, -0.05)	31.35	31.12	-0.23	(-0.43, -0.04)	31.35	31.10	-0.25	(-0.62, 0.12)	31.43	31.24	-0.20	(-0.86, 0.27)
Vaccination	14.38	29.61	15.23	(15.18, 15.28)	14.38	24.29	9.91	(9.87, 9.96)	14.38	21.88	7.50	(7.46, 7.54)	14.38	29.61	15.23	(15.18, 15.27)
Total																
Medical + Vaccination	770.80	780.61	9.81	(8.10, 11.77)	768.90	773.66	4.76	(3.10, 6.62)	770.80	773.40	2.60	(-104.13, 99.27)	1,638.48	1,649.51	11.02	(8.82, 13.37)
Medical + Non-Medical + Vaccination	802.15	811.72	9.57	(7.83, 11.61)	800.26	804.78	4.53	(2.82, 6.40)	802.15	804.50	2.35	(-104.74, 99.18)	1,669.92	1,680.74	10.82	(8.61, 13.25)
Life-Years (dis-counted)	10.3164	10.3183	0.0020	(-0.0047, 0.0078)	10.3166	10.3183	0.0018	(-0.0045, 0.0077)	10.3164	10.3183	0.0019	(-0.0044, 0.0085)	10.3159	10.3171	0.0012	(-0.0044, 0.0066)
QALY (discount-	7.0067	7.0079	0.0012	(-0.0032,	7.0068	7.0079	0.0010	(-0.0031,	7.0067	7.0078	0.0012	(-0.0028,	7.0065	7.0072	0.0007	(-0.0031,

ed)				0.0045)				0.0044)				0.0052)				0.0038)
**Healthcare System Perspective**																
Cost per Life-Year Gained	€ 5,006				€ 2,670				€ 1,336				€ 9,434			
Cost per QALY Gained	€ 8,474				€ 4,587				€ 2,247				€ 15,573			
**Societal Perspective**																
Cost per Life-Year Gained	€ 4,881				€ 2,542				€ 1,207				€ 9,264			
Cost per QALY Gained	€ 8,263				€ 4,367				€ 2,030				€ 15,292			

QALY: quality-adjusted life year

Note: Low-risk is specified as immunocompetent patients without any chronic medical conditions, moderate-risk describes immunocompetent patients with at least one chronic medical condition and high-risk represent immunocompromised/immunosuppressed patients, with or without chronic medical conditions (congenital or acquired).

Healthcare system perspective includes medical and vaccination costs; societal perspective includes medical, non-medical, and vaccination costs.
