# Supplementary material for: Estimating the cost-effectiveness of a sequential pneumococcal vaccination program for adults in Germany
Source: PLoS One. 2018 May 24;13(5):e0197905. doi: 10.1371/journal.pone.0197905 (PMC5967715; doi:10.1371/journal.pone.0197905)
Supplement: S9 Table — (PDF) [file pone.0197905.s010.pdf]

S9 Table. Expected lifetime disease-related cases, deaths, and costs in German adults ≥60 years (#5-#7)

**Scenario** (description)	**#5** (#1 with 64% NBP Effectiveness PPSV23)				**#6** (#1 with 50% revaccination rate)				**#7** (#2 with 50% revaccination rate)			
	**Cur-rent**	**Hypothet-ical**	**Δ**	**95% CI**	**Current**	**Hypothet-ical**	**Δ**	**95% CI**	**Current**	**Hypothet-ical**	**Δ**	**95% CI**
**Population-Level Results**												
No. of Cases												
IPD (in thou-sands)	24.502	24.495	-0.008	(-0.819, 0.792)	24.646	24.624	-0.023	(-1.064, 1.161)	24.510	24.468	-0.042	(-1.157, 1.075)
NBP (in millions)												
Requiring In-patient Care	6.471	6.446	-0.026	(-0.035, -0.013)	6.478	6.439	-0.039	(-0.051, -0.020)	6.477	6.440	-0.037	(-0.050, -0.019)
Requiring Outpatient Care	9.082	9.044	-0.038	(-0.053, -0.024)	9.103	9.081	-0.022	(-0.045, -0.005)	9.103	9.084	-0.019	(-0.040, -0.002)
No. of Deaths (in millions)	1.318	1.314	-0.004	(-0.009, 0.001)	1.317	1.310	-0.007	(-0.013, 0.000)	1.317	1.310	-0.007	(-0.013, 0.000)
Total Costs (in billions)												
Medical Care	16.357	16.279	-0.079	(-0.150, -0.006)	16.347	16.230	-0.116	(-0.155, -0.071)	16.368	16.256	-0.112	(-0.148, -0.065)
Non-Medical Care	0.676	0.673	-0.003	(-0.006, 0.000)	0.679	0.674	-0.005	(-0.010, -0.001)	0.679	0.674	-0.005	(-0.009, -0.001)
Vaccination	0.311	0.641	0.330	(0.329, 0.331)	0.312	0.641	0.330	(0.329, 0.331)	0.312	0.526	0.215	(0.214, 0.216)

Total												
Medical + Vaccination	16.669	16.920	0.251	(0.180, 0.324)	16.658	16.872	0.213	(0.176, 0.258)	16.679	16.782	0.103	(0.067, 0.149)
Medical + Non-Medical + Vaccination	17.345	17.593	0.248	(0.176, 0.320)	17.337	17.545	0.208	(0.171, 0.255)	17.358	17.456	0.098	(0.062, 0.146)
**Patient-Level Results**												
Total Costs												
Medical Care	755.14	751.51	-3.63	(-6.93, -0.28)	754.66	749.28	-5.37	(-7.14, -3.27)	755.64	750.47	-5.17	(-6.81, -3.02)
Non-Medical Care	31.23	31.07	-0.16	(-0.28, -0.02)	31.34	31.10	-0.25	(-0.44, -0.05)	31.34	31.11	-0.23	(-0.42, -0.04)
Vaccination	14.38	29.61	15.23	(15.20, 15.26)	14.38	29.61	15.23	(15.19, 15.27)	14.38	24.29	9.91	(9.87, 9.96)
Total												
Medical + Vaccination	769.52	781.12	11.60	(8.30, 14.95)	769.04	778.89	9.86	(8.11, 11.93)	770.02	774.76	4.74	(3.10, 6.86)
Medical + Non-Medical + Vaccination	800.75	812.19	11.44	(8.14, 14.78)	800.38	809.99	9.61	(7.88, 11.77)	801.36	805.87	4.51	(2.87, 6.75)
Life-Years (discounted)	10.3175	10.3182	0.0007	(-0.0040, 0.0046)	10.3164	10.3182	0.0018	(-0.0046, 0.0076)	10.3166	10.3182	0.0016	(-0.0045, 0.0071)
QALY (discounted)	8.6846	8.6852	0.0006	(-0.0032, 0.0036)	7.0067	7.0078	0.0011	(-0.0032, 0.0046)	7.0068	7.0078	0.0009	(-0.0031, 0.0042)

**Healthcare System Perspective**			
Cost per Life-Year Gained	€ 16,345	€ 5,404	€ 2,921
Cost per QALY Gained	€ 20,720	€ 9,135	€ 5,149
**Societal Perspective**			
Cost per Life-Year Gained	€ 16,121	€ 5,270	€ 2,777
Cost per QALY Gained	€ 20,437	€ 8,908	€ 4,896

QALY: quality-adjusted life year

Note: Low-risk is specified as immunocompetent patients without any chronic medical conditions, moderate-risk describes immunocompetent patients with at least one chronic medical condition and high-risk represent immunocompromised/immunosuppressed patients, with or without chronic medical conditions (congenital or acquired). Healthcare system perspective includes medical and vaccination costs; societal perspective includes medical, non-medical, and vaccination costs.
