# Supplementary figures and images for: Estimating the cost-effectiveness of a sequential pneumococcal vaccination program for adults in Germany
Source: PLoS One. 2018 May 24;13(5):e0197905. doi: 10.1371/journal.pone.0197905 (PMC5967715; doi:10.1371/journal.pone.0197905)

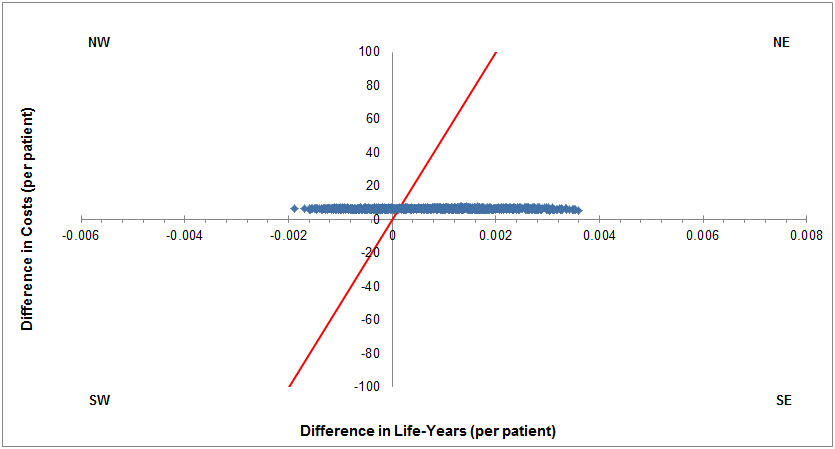

Supplement: S1 Fig — (TIF) [file pone.0197905.s011.tif]

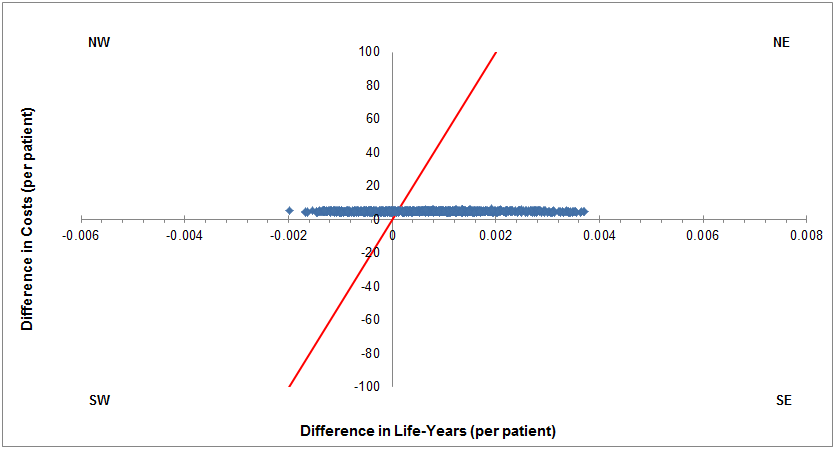

Supplement: S2 Fig — (TIF) [file pone.0197905.s012.tif]

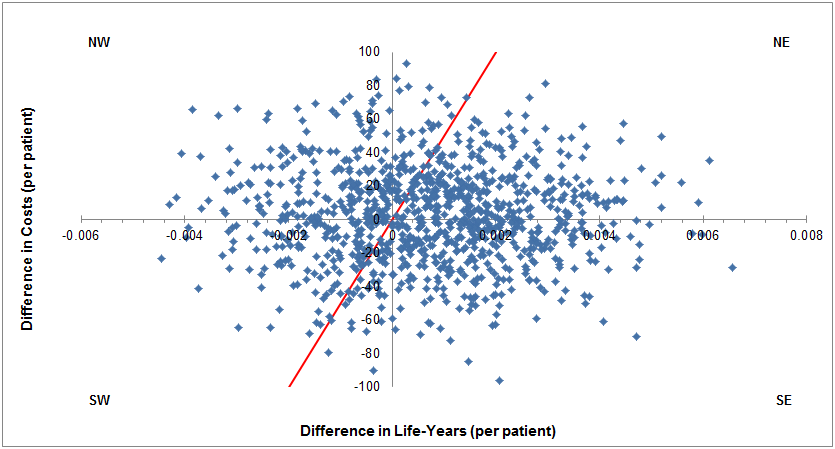

Supplement: S3 Fig — (TIF) [file pone.0197905.s013.tif]

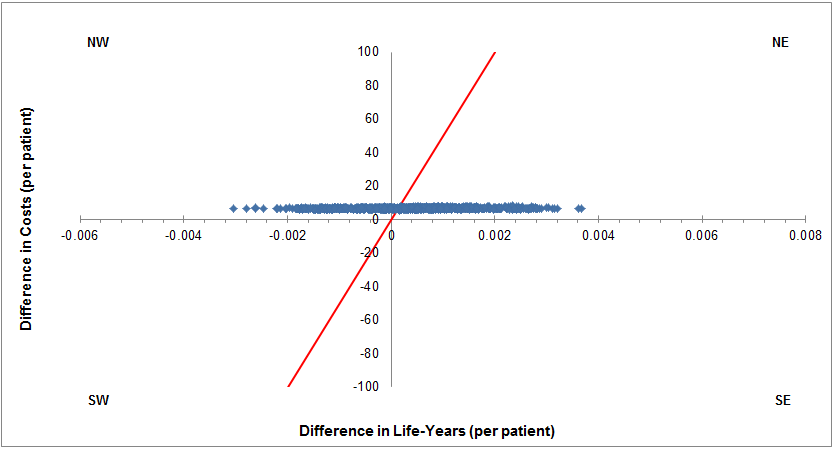

Supplement: S4 Fig — (TIF) [file pone.0197905.s014.tif]

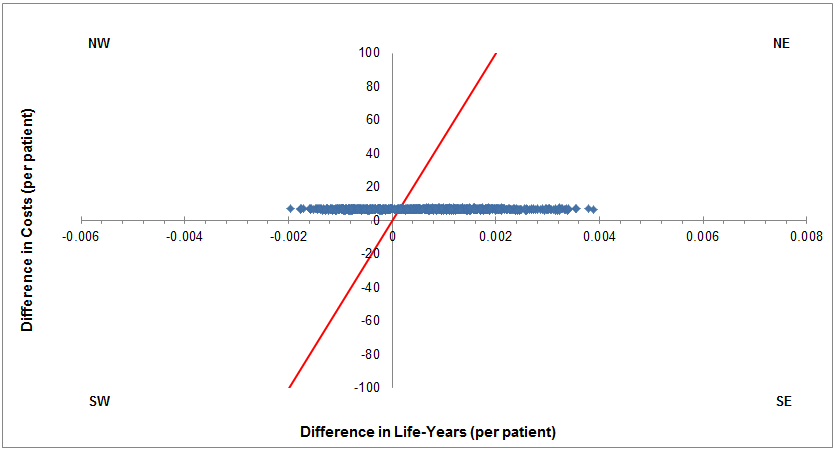

Supplement: S5 Fig — (TIF) [file pone.0197905.s015.tif]

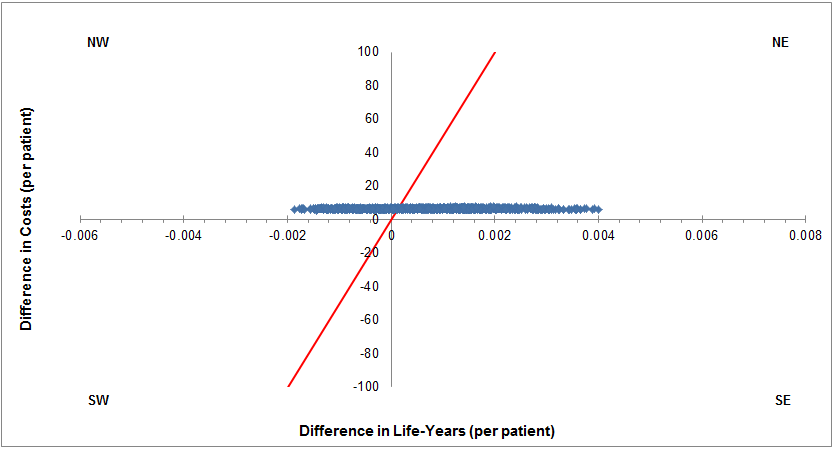

Supplement: S6 Fig — (TIF) [file pone.0197905.s016.tif]

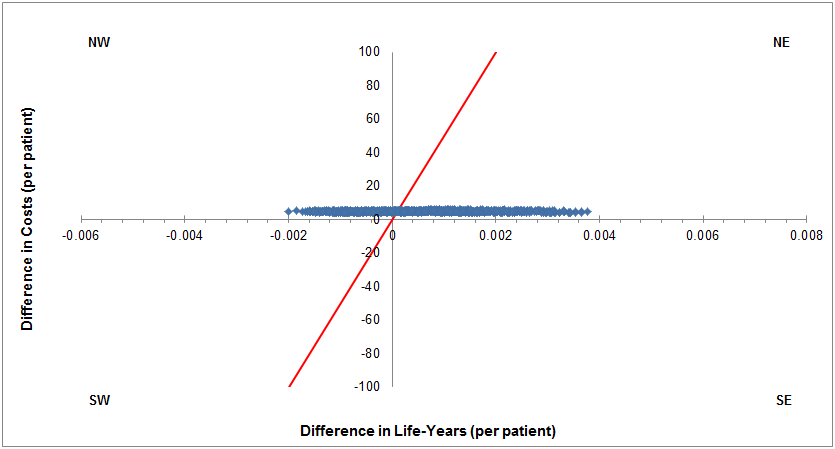

Supplement: S7 Fig — (TIF) [file pone.0197905.s017.tif]
